# Coping with alpine habitats: genomic insights into the adaptation strategies of *Triplostegia glandulifera* (Caprifoliaceae)

**DOI:** 10.1093/hr/uhae077

**Published:** 2024-05-01

**Authors:** Jian Zhang, Kai-Lin Dong, Miao-Zhen Ren, Zhi-Wen Wang, Jian-Hua Li, Wen-Jing Sun, Xiang Zhao, Xin-Xing Fu, Jian-Fei Ye, Bing Liu, Da-Ming Zhang, Mo-Zhu Wang, Gang Zeng, Yan-Ting Niu, Li-Min Lu, Jun-Xia Su, Zhong-Jian Liu, Pamela S Soltis, Douglas E Soltis, Zhi-Duan Chen

**Affiliations:** State Key Laboratory of Plant Diversity and Specialty Crops & Key Laboratory of Systematic and Evolutionary Botany, Institute of Botany, Chinese Academy of Sciences, Beijing 100093, China; China National Botanical Garden, Beijing 100093, China; State Key Laboratory of Plant Diversity and Specialty Crops & Key Laboratory of Systematic and Evolutionary Botany, Institute of Botany, Chinese Academy of Sciences, Beijing 100093, China; China National Botanical Garden, Beijing 100093, China; University of Chinese Academy of Sciences, Beijing 100049, China; Key Laboratory of Resource Biology and Biotechnology in Western China, Ministry of Education, College of Life Sciences, Northwest University, Xi'an 710069, China; PubBio-Tech Services Corporation, Wuhan 430070, China; Biology Department, Hope College, Holland, MI 49423, USA; State Key Laboratory of Plant Diversity and Specialty Crops & Key Laboratory of Systematic and Evolutionary Botany, Institute of Botany, Chinese Academy of Sciences, Beijing 100093, China; China National Botanical Garden, Beijing 100093, China; University of Chinese Academy of Sciences, Beijing 100049, China; PubBio-Tech Services Corporation, Wuhan 430070, China; College of Life Sciences, Northwest Normal University, Lanzhou 730070, China; School of Ecology, Shenzhen Campus of Sun Yat-sen University, Shenzhen 518107, China; State Key Laboratory of Plant Diversity and Specialty Crops & Key Laboratory of Systematic and Evolutionary Botany, Institute of Botany, Chinese Academy of Sciences, Beijing 100093, China; China National Botanical Garden, Beijing 100093, China; Sino-Africa Joint Research Center, Chinese Academy of Sciences, Wuhan 430074, China; State Key Laboratory of Plant Diversity and Specialty Crops & Key Laboratory of Systematic and Evolutionary Botany, Institute of Botany, Chinese Academy of Sciences, Beijing 100093, China; China National Botanical Garden, Beijing 100093, China; State Key Laboratory of Plant Diversity and Specialty Crops & Key Laboratory of Systematic and Evolutionary Botany, Institute of Botany, Chinese Academy of Sciences, Beijing 100093, China; China National Botanical Garden, Beijing 100093, China; Xishuangbanna Tropical Botanical Garden, Chinese Academy of Sciences, Menglun 666303, China; State Key Laboratory of Plant Diversity and Specialty Crops & Key Laboratory of Systematic and Evolutionary Botany, Institute of Botany, Chinese Academy of Sciences, Beijing 100093, China; China National Botanical Garden, Beijing 100093, China; State Key Laboratory of Plant Diversity and Specialty Crops & Key Laboratory of Systematic and Evolutionary Botany, Institute of Botany, Chinese Academy of Sciences, Beijing 100093, China; China National Botanical Garden, Beijing 100093, China; School of Life Science, Shanxi Normal University, Taiyuan 030031, China; Key Laboratory of National Forestry and Grassland Administration for Orchid Conservation and Utilization, Fujian Agriculture and Forestry University, Fuzhou 350002, China; Florida Museum of Natural History, University of Florida, Gainesville, FL 32611, USA; Florida Museum of Natural History, University of Florida, Gainesville, FL 32611, USA; Department of Biology, University of Florida, Gainesville, FL 32611-7800, USA; State Key Laboratory of Plant Diversity and Specialty Crops & Key Laboratory of Systematic and Evolutionary Botany, Institute of Botany, Chinese Academy of Sciences, Beijing 100093, China; China National Botanical Garden, Beijing 100093, China; Sino-Africa Joint Research Center, Chinese Academy of Sciences, Wuhan 430074, China

## Abstract

How plants find a way to thrive in alpine habitats remains largely unknown. Here we present a chromosome-level genome assembly for an alpine medicinal herb, *Triplostegia glandulifera* (Caprifoliaceae), and 13 transcriptomes from other species of Dipsacales. We detected a whole-genome duplication event in *T. glandulifera* that occurred prior to the diversification of Dipsacales. Preferential gene retention after whole-genome duplication was found to contribute to increasing cold-related genes in *T. glandulifera*. A series of genes putatively associated with alpine adaptation (e.g. *CBF*s, *ERF-VII*s, and *RAD51C*) exhibited higher expression levels in *T. glandulifera* than in its low-elevation relative, *Lonicera japonica*. Comparative genomic analysis among five pairs of high- vs low-elevation species, including a comparison of *T. glandulifera* and *L. japonica*, indicated that the gene families related to disease resistance experienced a significantly convergent contraction in alpine plants compared with their lowland relatives. The reduction in gene repertory size was largely concentrated in clades of genes for pathogen recognition (e.g. *CNL*s, *prRLP*s, and XII *RLK*s), while the clades for signal transduction and development remained nearly unchanged. This finding reflects an energy-saving strategy for survival in hostile alpine areas, where there is a tradeoff with less challenge from pathogens and limited resources for growth. We also identified candidate genes for alpine adaptation (e.g. *RAD1*, *DMC1*, and *MSH3*) that were under convergent positive selection or that exhibited a convergent acceleration in evolutionary rate in the investigated alpine plants. Overall, our study provides novel insights into the high-elevation adaptation strategies of this and other alpine plants.

## Introduction

Plants have adapted to a wide array of habitats, including many extreme environments, including high elevations, deserts, and polar regions. Among these, alpine ecosystems are a matter of great concern because they are usually highly biodiverse and highly sensitive to climate change [1, 2]. High-elevation habitats are characterized by lower temperature, lower oxygen, and higher ultraviolet (UV) radiation compared with most low-elevation habitats. In response to these environmental challenges, alpine plants generally develop distinctive morphological traits, such as a dwarf stature, a cushion form, and a reduction in leaf size, which could result from convergent adaptive evolution [3, 4]. Recently, various omics technologies, such as genome sequencing, transcriptomics, and proteomics, have advanced research on the adaptation of alpine plants [5–8]. The molecular mechanisms of alpine adaptation have been gradually revealed via the detection of positively selected genes (PSGs), fast-evolving genes (FEGs), expanded gene families, and differentially expressed genes (DEGs) [5–8]. Candidate genes discovered to date for high-elevation adaptation are involved in different functional pathways, especially DNA repair, UV-B tolerance, and cold tolerance [5, 9]. Approximately 100 flowering plant families have alpine representatives [3], and recent research into their adaptations has only revealed the tip of the iceberg. Comparative genomics studies for high- vs low-elevation species that explore the underlying genomic convergence of alpine adaptation among different families are very rare [5].

Due to global warming, the geographic range of some alpine plants is decreasing [10, 11]. *Triplostegia glandulifera* Wall. ex DC. is a typical alpine plant, of which the distribution is continuously shrinking, because it is highly susceptible to changes in habitat and climate [12]. *Triplostegia glandulifera* is mainly distributed in the Himalaya–Hengduan Mountain region and Taiwan region, Central Sulawesi, and New Guinea, with the highest elevation recorded at 4000 m [13]. Its root is a key component of traditional Chinese medicine used for liver protection and the treatment of kidney diseases. *Triplostegia glandulifera* belongs to Caprifoliaceae, the honeysuckle family (Dipsacales), which includes many cold-adapted species from high elevations and high latitudes [14, 15]. The genome of the low-elevation species *Lonicera japonica* (Caprifoliaceae) has recently been published, making an omics comparison between high- and low-elevation Caprifoliaceae species possible [16]. Therefore, *T. glandulifera* presents a promising candidate for investigating the adaptive evolution and environmental sensitivity of high-elevation plants. Understanding its adaptation strategies will be helpful for its cultivation and conservation. However, as of now, no relevant research has been published on *T. glandulifera* or any other Caprifoliaceae.

In this study we report a chromosome-level genome assembly of *T. glandulifera* and present 13 transcriptomes from other species of Dipsacales. Through comparative genomic analyses involving Dipsacales species or pairs of high- vs low-elevation species from different angiosperm families, we characterize genomic features, whole-genome duplications (WGDs), differential gene expression, gene family evolution, and adaptive signals in gene sequences. We find that preferential gene retention after WGD increased the copy number of cold-related genes in *T. glandulifera*. We also detect many genes with higher expression levels in *T. glandulifera* compared with its low-elevation relative, *L. japonica*, a series of which are putatively associated with alpine adaptation. We document the convergent contraction of gene families related to disease resistance in *T. glandulifera* and other investigated alpine plants when compared with their lowland relatives. We identify a set of FEGs and PSGs in the investigated alpine plants, many of which might be involved in alpine adaptation, such as DNA repair, cold response, and hypoxia response. Our findings provide novel insights into the adaptation strategies of alpine plants and also have implications for the conservation of these environmentally sensitive plants.

## Results

### Genome sequencing, assembly, and annotation

**Figure 1:**
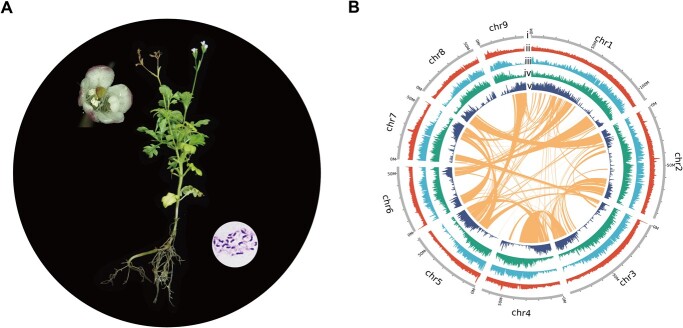
*Triplostegia glandulifera* plant and its genome. **A** Chromosomes (2*n* = 18) (right panel) and morphology of *T. glandulifera* with the flower (left panel), leaf, stem and root displayed. **B** Landscape of the *T. glandulifera* genome. Tracks i–v represent nine pseudochromosomes (Mb), GC content, repeat element density, LTR density, and gene density, respectively. The inner curve lines indicate collinear gene blocks.

The genome size of *T. glandulifera* (Fig. 1A) was estimated to be 719.94 Mb in the *k*-mer analysis (Supplementary Data Fig. S1) and 668.05 Mb in the flow cytometry test (Supplementary Data Fig. S2). We used a total of 124.17 Gb Illumina, 98.30 Gb PacBio, 95.89 Gb 10x Genomics, and 149.61 Gb Hi-C sequencing reads for the *de novo* assembly of the *T. glandulifera* genome (Supplementary Data Table S1). After the primary assembly, correction, polishing, and scaffolding (see Materials and methods section), we obtained a final assembly of 680.38 Mb (contig N50 = 1.81 Mb, scaffold N50 = 66.68 Mb) within 1233 scaffolds (Supplementary Data Tables S2 and S3). The majority (~91.25%) of the genome was anchored to nine pseudomolecules corresponding to the chromosomes (*n* = 9, Fig. 1B), with the sizes ranging from 36.35 to 110.58 Mb (Supplementary Data Fig. S3, Supplementary Data Table S4). The mapping rate of the assembled genome reached 98.14% based on the Illumina short reads (Supplementary Data Table S5). The genome and protein BUSCO [17] scores were 97.4 and 95.4%, respectively (Supplementary Data Table S6). Approximately 94.47% of the *T. glandulifera* transcriptome data can be mapped back to the assembled genome (Supplementary Data Table S7).

Repeat sequences comprised 62.88% of the *T. glandulifera* genome (Supplementary Data Table S8), with transposable elements (TEs) being the major component (Supplementary Data Table S9). Long terminal repeats (LTRs) were the most abundant type of TEs, covering 52.09% of the genome (Supplementary Data Table S9). Using a combination of *de novo*, homology-based, and RNA sequence-based predictions, we identified 32 123 protein-coding genes, with an average length of 3351 bp (Supplementary Data Table S10). Notably, 95.53% of the protein-coding genes were annotated in at least one of the six functional databases (Supplementary Data Table S11). Additionally, we identified 1427 miRNA, 755 tRNA, 6905 rRNA, and 552 snRNA genes (see Materials and methods section and Supplementary Data Table S12).

### Phylogenomics and whole-genome duplication

We conducted the phylogenomic and molecular dating analyses based on 106 single-copy genes derived from the genome sequences of *T. glandulifera*, *L. japonica*, *Daucus carota* (Apiaceae), and the transcriptome sequences from 15 other species of Dipsacales covering Viburnaceae and all subfamilies in Caprifoliaceae, including 13 newly sequenced species and two species for which transcriptomes were previously published (see Materials and methods section, Supplementary Data Fig. S4 and Supplementary Data Tables S13 and S14). *Triplostegia glandulifera* was included in subfamily Dipsacoideae of Caprifoliaceae with strong support for the topology [*T. glandulifera* (*Scabiosa tschiliensis*, *Dipsacus fullonum*)] (Supplementary Data Fig. S5).

**Figure 2:**
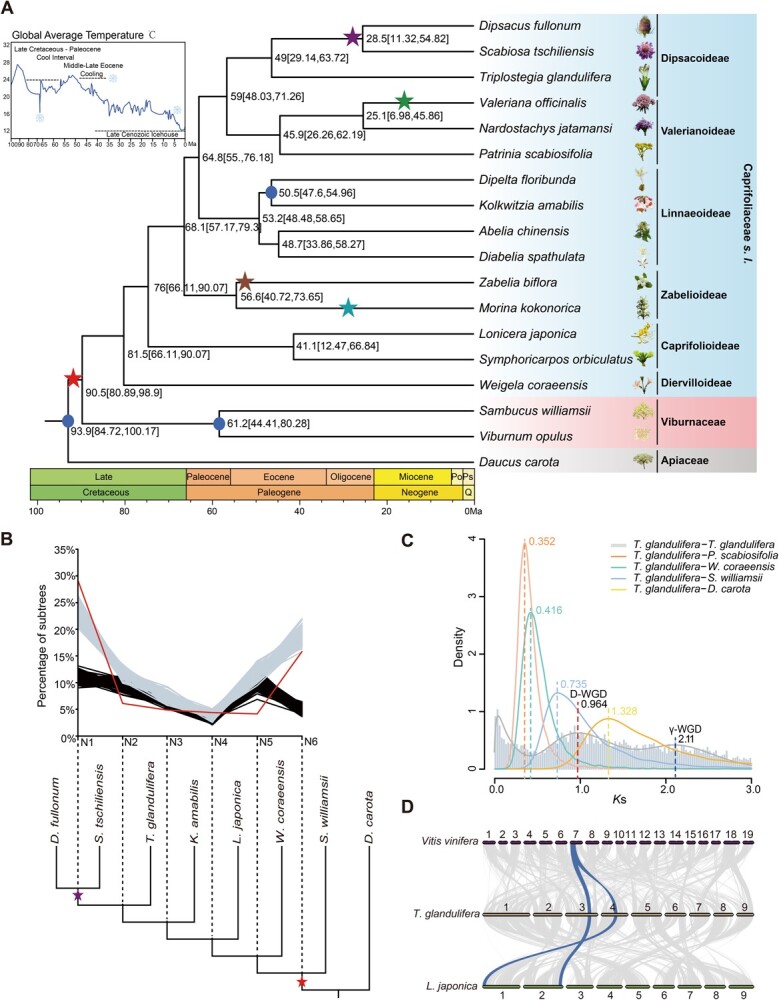
Time tree of Dipsacales and genome evolution of *T. glandulifera*. **A** Chronogram showing divergence times and WGD events in Dipsacales (including Viburnaceae and Caprifoliaceae). The asterisks show predicated WGDs in Dipsacales, and different colors correspond to different *K*_s_ peaks in Supplementary Data Fig. S6. The blue dots indicate the placement of the calibrations for molecular dating. The blue curve on the top left of the tree shows the fluctuations of global average temperature over the past 100 Ma [19]. The species photos were provided by Liu Bing, Kai-Lin Dong, Jian-Fei Ye, and Li-Xin Zhou. **B** Multi-tAxon Paleopolyploidy Search (MAPS) on the phylogeny. The red line indicates the percentage of subtrees that contained a gene duplication shared by descendant species at each node. The null simulations and positive simulations are displayed by black lines and gray lines, respectively. The red and purple asterisks represent the D-WGD in Dipsacales and another recent WGD shared by *D. fullonum* and *S. tschiliensis*, respectively. **C***K*_s_ distribution from paralogs of *T. glandulifera* (gray line) and orthologs of *T. glandulifera* vs each of the four species (colorful lines) (*P. scabiosifolia*, *W. coraeensis*, *S. williamsii*, and *D. carota*). D-WGD specific to Dipsacales and γ-WGD shared by all core eudicots are highlighted. **D** Synteny blocks among chromosomes of *T. glandulifera*, *L. japonica*, and *V. vinifera*. Synteny analysis suggested the 2:1 syntenic relationship between *T. glandulifera* and *V. vinifera*, and 1:1 between *T. glandulifera* and *L. japonica* (blue highlighted).

We estimated the crown age of Dipsacales to be ~90.5 Ma (95% highest posterior density (HPD) interval 80.89–98.9 Ma) (Fig. 2A), suggesting that Caprifoliaceae and Viburnaceae originated during a period of global cooling in the Late Cretaceous [18, 19]. The diversification time of Caprifoliaceae was estimated to be ~81.5 Ma (95% HPD interval 66.11–90.07 Ma) (Fig. 2A). The stem age of *Triplostegia*, based on our species sampling, was dated to ~49 Ma (95% HPD interval 29.14–63.72 Ma) (Fig. 2A).

Our various analyses including synonymous substitutions per synonymous site (*K*_s_) age distribution, phylogenetic approaches, and synteny assessments (see Materials and methods section) suggested at least five WGDs across the Dipsacales phylogeny (Fig. 2, Supplementary Data Figs S6–S8). One of these WGDs is shared by all investigated species of Dipsacales, another is shared at least by *D. fullonum* and *S. tschiliensis*, and the remaining three are specific to *Valeriana officinalis*, *Zabelia biflora*, and *Morina kokonorica*, respectively (Fig. 2, Supplementary Data Figs S6–S8). The *K*_s_ distribution for the *T. glandulifera* paralogs showed a signature peak for the WGD event (*K*_s_ 0.964) following the γ-WGD (*K*_s_ 2.11) shared by all core eudicots [20] (Fig. 2C). The *K*_s_ value of paralogs in *T. glandulifera* was higher than that of orthologs shared by *T. glandulifera* and other species of Dipsacales but lower than that shared by *T. glandulifera* and *D. carota* (Fig. 2C). Combined with *K*_s_ distributions for 16 other species of Dipsacales, this duplication in *T. glandulifera* seems to be shared by all Dipsacales species, herein named D-WGD (Fig. 2C, Supplementary Data Fig. S6). Additional signature peaks for recent WGDs were also observed in *D. fullonum*, *S. tschiliensis*, *V. officinalis*, *Z. biflora*, and *M. kokonorica* (Supplementary Data Fig. S6). Through the Multi-tAxon Paleopolyploidy Search (MAPS) analysis, the Dipsacales-specific D-WGD was further confirmed, while the recent WGDs in *D. fullonum* and *S. tschiliensis* were found to be shared (Fig. 2B, Supplementary Data Table S15). Intra- and intergenomic syntenic analyses revealed a 2:1 syntenic depth ratio between *T. glandulifera* and *Vitis vinifera* [21], as well as between *L. japonica* and *V. vinifera*, and a 1:1 syntenic relationship between *T. glandulifera* and *L. japonica* (Fig. 2D, Supplementary Data Figs S7–S8). These results provide the syntenic evidence for the D-WGD event shared by *T. glandulifera* and *L. japonica*. The age of the D-WGD was estimated to be ~91.97 Ma, just before the diversification of Dipsacales during the Late Cretaceous (Fig. 2A, Supplementary Data Table S16).

In the *T. glandulifera* genome we identified 24 840 (77.33%) duplicated genes derived from five different modes of duplication (WGD; DSD, dispersed duplication; TRD, transposed duplication; TD, tandem duplication; PD, proximal duplication) using DupGen_finder [22]. WGD was the most prevalent type of gene duplication in *T. glandulifera*, as well as in *L. japonica* (Supplementary Data Fig. S9, Supplementary Data Table S22). The *T. glandulifera* WGD gene sets showed a significant enrichment in biological processes linked to signal response (such as response to cold, response to auxin, and response to gibberellin) and plant development (such as floral organ development, leaf development, and shoot system development) in the functional enrichment analyses (Supplementary Data Figs S10 and S11, Supplementary Data Tables S23–S32).

### Gene evolution and differential expression

**Figure 3:**
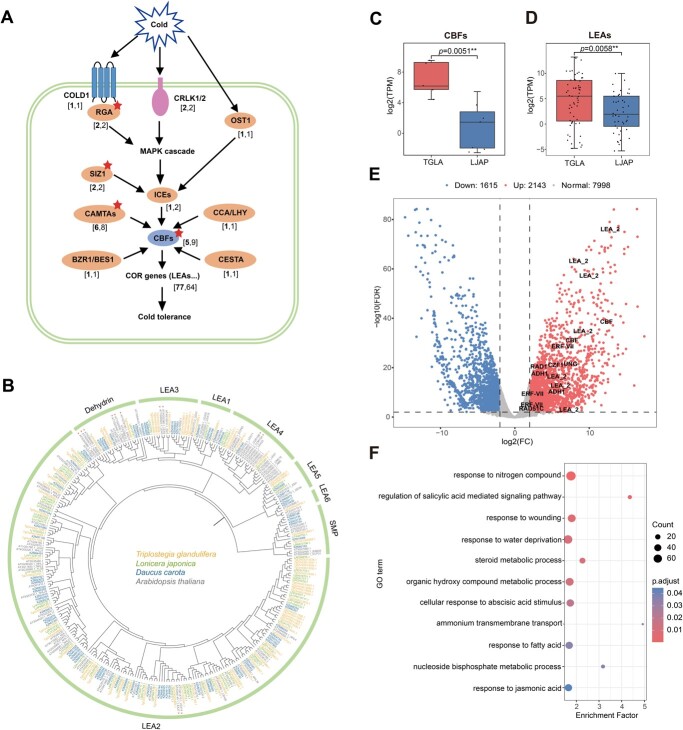
Gene evolution and differential expression. **A** Overview of the regulation of the CBF-dependent cold signaling pathway. The numbers of corresponding genes in *T. glandulifera* and *L. japonica* are shown in parentheses in the format of [***T. glandulifera***, *L. japonica*]. The red asterisks indicate the retention of duplicated copies after the D-WGD event in *T. glandulifera*. **B** Phylogenetic tree of *LEA* genes from *T. glandulifera*, *L. japonica*, *D. carota*, and *A. thaliana*. The red asterisks indicate the well-known *Arabidopsis* CORs. **C** Gene expression boxplot for *CBF* genes in *T. glandulifera* and *L. japonica* (**P < 0.01). **D** Gene expression boxplot for *LEA* genes in *T. glandulifera* and *L. japonica* (**P < 0.01). **E** DEGs in *T. glandulifera* compared with *L. japonica*. Red dots represent significantly upregulated genes, blue dots represent significantly downregulated genes, and gray dots represent non-significant ones. Some upregulated genes with potential functions in alpine adaptation are shown (Supplementary Data Table S18). **F** The significantly enriched GO terms for upregulated genes in *T. glandulifera* compared with *L. japonica*. The size of the circles shows the number of genes in one GO term. The color of the circles displays the statistical significance of enriched GO terms.

Cold stress is a major abiotic stress that significantly impacts plant growth, survival, and geographical distribution, especially in alpine plants [3, 23]. Among various cold-tolerance mechanisms, the CBF (C-repeat binding transcription factor)-dependent cold signaling pathway is considered to be the most well-known; it is highly conserved among various plant species [23, 24]. In this pathway, CBFs are the key transcription factors [24]. We found that WGD-derived genes in *T. glandulifera* enriched the number of genes that function in response to cold (Supplementary Data Fig. S10); we further surveyed the CBFs and 10 well-known positive regulators for cold tolerance in the CBF-dependent cold signaling pathway in Dipsacales and investigated the contribution of WGD in the evolution of these genes [23, 24] (Fig. 3A, Supplementary Data Figs S12–S22).

Phylogenetic analysis of *CBF*s suggested that the D-WGD event resulted in two clades (*DipsCBFa* and *DipsCBFb*) in Dipsacales (Supplementary Data Fig. S12). In the *DipsCBFa* and *DipsCBFb* clades, *CBF*s further expanded by TD events, leading to five homologs in *T. glandulifera* and nine in *L. japonica* (Fig. 3A, Supplementary Data Fig. S12). Gene expression boxplots revealed that the average expression levels of *CBF*s in *T. glandulifera* were significantly higher than those in *L. japonica* (*P* = 0.0051) (Fig. 3C and Supplemental Data Fig. S12). Phylogenetic analyses also suggest that genes encoding RGAs (rice G-protein α subunits), SUMO E3 ligase SIZ1 (SAP and Miz1), ICEs (inducers of CBF expression), and CAMTAs (calmodulin-binding transcription activators) were duplicated into multiple copies through the D-WGD event in Dipsacales (Supplementary Data Figs S15 and S17–S19). Cold-responsive genes (CORs) are regulated under CBFs and directly cope with cold stress, with most of these genes encoding late embryogenesis abundant (LEA) proteins (Fig. 3A) [25]. *Triplostegia glandulifera* had 77 LEA proteins, while *L. japonica* had 64 (Fig. 3A and B, Supplementary Data Table S33), and the average expression levels of *LEA*s in *T. glandulifera* were significantly higher than those in *L. japonica* (*P* = 0.0058) (Fig. 3D).

Furthermore, we conducted pairwise comparisons between *T. glandulifera* and *L. japonica* leaf transcriptomes (Supplementary Data Table S17) to identify DEGs (Fig. 3E and F, Supplementary Data Table S18). We identified a total of 3758 DEGs (2143 upregulated and 1615 downregulated genes) in *T. glandulifera* compared with *L. japonica* (Fig. 3E). Among the upregulated genes, the functional enrichment terms were significantly overrepresented in categories such as ‘response to nitrogen compound’, ‘response to wounding’, and ‘response to water deprivation’ (Fig. 3F, Supplementary Data Tables S34 and S35). More importantly, we discovered a series of upregulated genes in *T. glandulifera* with potential functions in alpine adaptation, including genes related to cold tolerance (e.g. *CBF*s, *LEA*s, *CZF1*) [23–25], hypoxia response (*ERF-VII*s and *ADH1*s) [26], and DNA repair (e.g. *RAD1*, *RAD51C*, *UNG*) [27, 28] (Supplementary Data Table S18).

### Molecular convergence

#### Convergent contraction of gene families related to disease resistance

**Figure 4:**
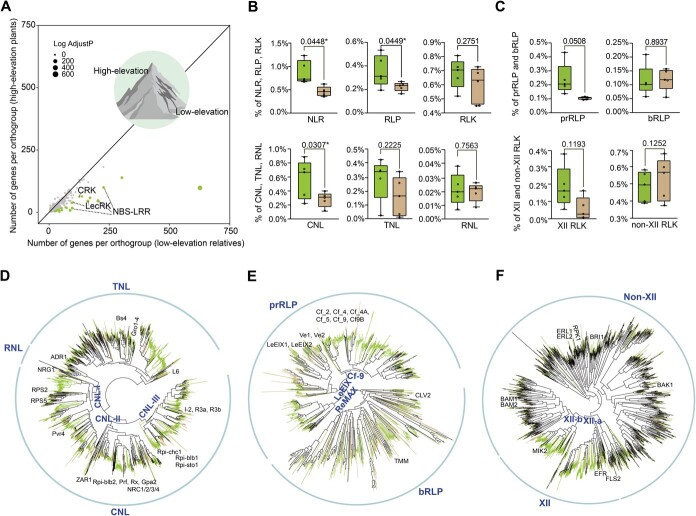
Convergent contraction of gene families related to disease resistance in *T. glandulifera* and other alpine plants. **A** Scatter plot showing significantly convergently expanded (yellow-brown)/contracted (green) gene families in high-elevation plants compared with their low-elevation relatives. The circle sizes stand for log_2_(*P*-adjust), where *P*-adjust is the *P*-value of the binomial test adjusted for multiple testing. Boxplots for gene percentages of plant immune receptors between high-elevation plants (yellow-brown) and low-elevation ones (green): NBS-LRRs (NLRs), LRR-RLPs (RLPs), LRR-RLKs (RLKs) in the upper panel, and CNL, TNL, and RNL clades of NLRs in the lower panel (**B**); the pathogen-responsive RLPs (prRLPs) and basal RLPs (bRLPs) of RLPs in the upper panel, and the XII- and non-XII clades of RLKs in the lower panel (**C**). The percentage is the number of identified genes/number of searched genes. A paired sample *t*-test was used to analyze significant differences between the groups (*P < 0.05). **D** Phylogenetic tree of *NLR* genes from *T. glandulifera*, *L. japonica*, four other pairs of investigated plants, *A. thaliana,* and species with known function NLRs. **E** Phylogenetic tree of *RLP* genes from *T. glandulifera*, *L. japonica*, four other pairs of investigated plants, *A. thaliana,* and species with known function RLPs. **F** Phylogenetic tree of *RLK* genes from *T. glandulifera*, *L. japonica*, four other pairs of investigated plants, and *A. thaliana*. **E**–**F** The yellow-brown lines label genes from high-elevation plants, the green lines label genes from low-elevation plants, and the black lines label known function genes and genes from *A. thaliana*. The well-known NLRs, RLPs, and RLKs are shown with names.

Gene family expansion and contraction analysis using CAFÉ [29] (see Materials and methods section and Supplementary Data Tables S19 and S20) indicated that the expanded gene families in *T. glandulifera* were significantly enriched in metabolic functions, while contracted ones were significantly enriched in plant–pathogen interaction pathways (Supplementary Data Fig. S23, Supplementary Data Tables S36–S39). To investigate whether convergent changes in gene copy number occurred in distantly related alpine plants, we detected the convergent expansion or contraction of gene families in high-elevation plants compared with their low-elevation relatives from five different families: Caprifoliaceae (*T. glandulifera* vs *L. japonica*), Asteraceae (*Erigeron breviscapus* vs *E. canadensis*), Ericaceae (*Rhododendron williamsianum* vs *R. ovatum*), Brassicaceae (*Crucihimalaya himalaica* vs *Capsella rubella*), and Salicaceae (*Salix brachista* vs *S. viminalis*) (see Materials and methods section and Supplementary Data Table S21). All five pairs of plants underwent independent lineage-specific WGDs, without the effect of species-specific WGD (Fig. 2C, Supplementary Data Fig. S24). Only one gene family, belonging to the histidine phosphatase superfamily, displayed a striking expansion in all of these alpine plant genomes (Supplementary Data Table S40). In contrast, 28 gene families showed a significantly convergent contraction, including several genes associated with plant immunity (Fig. 4A, Supplementary Data Table S40). Examples include intracellular nucleotide-binding leucine-rich repeat receptors (NBS-LRRs, NLRs for short), the largest gene family for plant disease resistance [30], the L-type lectin receptor-like kinases (LecRKs) for plant innate immunity [31], and the cysteine-rich receptor-like kinases (CRKs) for disease resistance and programmed cell death [32].

Plants have evolved a two-tier immune system to recognize and activate defense against pathogen infections, including pattern-triggered immunity (PTI) and effector-triggered immunity (ETI) [33]. The initiation of PTI and ETI needs cell-surface and intracellular immune receptors, respectively (Supplementary Data Fig. S25). Leucine-rich repeat-domain-containing receptor-like kinases (LRR-RLKs, RLKs for short) and receptor-like proteins (LRR-RLPs, RLPs for short) without the protein kinase domain are two major components of cell-surface immune receptors that recognize pathogen-associated molecular patterns (PAMPs) and activate PTI [34]. NLRs serve as intracellular immune receptors that recognize pathogen-secreted effectors, activate ETI, and induce a hypersensitive cell death response [34].

We investigated the canonical *NLR*, *RLP*, and *RLK* genes in the above-mentioned five pairs of high- vs low-elevation species (Fig. 4B–F, Supplementary Data Figs S26–S29; Supplementary Data Tables S41–S54). We identified 2395 *NLR *genes, 993 *RLP* genes, and 2177 *RLK *genes among all of the genomes investigated here (Supplementary Data Tables S46, S49 and S52). To exclude the effect of WGDs and proteome sizes, we normalized the gene repertory size by using percentages (number of identified genes/number of searched genes). The alpine plants showed a convergent contraction in the size of these three gene families compared with their lowland relatives, especially for *NLR*s (*P* = 0.0448) and *RLP*s (*P* = 0.0449) (Fig. 4B, Supplementary Data Tables S46, S49 and S52). Our phylogenetic tree revealed three clades of estimated NLRs: CC-NBS-LRR (CNL), TIR-NBS-LRR (TNL), and RPW8-NBS-LRR (RNL) (Fig. 4D, Supplementary Data Fig. S26, Supplementary Data Tables S46–S48), consistent with the presence of different conserved domains at the N-terminus [35]. The CNL clade was further divided into three subclades, CNL-I (e.g. *RPS2* and *RPS5*), CNL-II (e.g. *ZAR1* and *NRC4*), and CNL-III (e.g. *I2* and *R3a*) (Fig. 4D, Supplementary Data Fig. S26), which correspond to different subclasses of CC domains, CC_CAN_, CC_EDVID_, and CC_I2-like_, respectively [36]. The co-contraction trend in high-elevation plants was also clearly shown in the CNLs and TNLs (Fig. 4B), particularly in the CNLs (*P* = 0.0307), with CNL-I (*P* = 0.0559) and CNL-II (*P* = 0.0625) subclades slightly below the level of significance (Supplementary Data Fig. S29). In contrast, the percentage of RNLs was similar between high- and low-elevation plants (Fig. 4B). Based on our phylogenetic tree, the RLPs grouped into two distinct clades: pathogen-responsive RLPs (prRLPs) and basal RLPs (bRLPs) clades, and the prRLPs were further divided into Cf-9, LeEIX, and ReMAX subclades (Fig. 4E, Supplementary Data Fig. S27, Supplementary Data Tables S49–S51), in agreement with previous studies [37, 38]. The prRLPs play a crucial role in the immune response, while bRLPs primarily regulate plant growth and development in *Arabidopsis thaliana* [38]. We observed a nearly significant trend of contraction in the prRLPs clade (*P* = 0.0508) and the Cf-9 subclade (*P* = 0.0534) in high-elevation plants (Fig. 4C, Supplementary Data Fig. S29), while the proportion of bRLPs remained nearly unchanged between high- and low-elevation plants (Fig. 4C). The RLKs include 20 subgroups (Fig. 4F, Supplementary Data Fig. S28, Supplementary Data Tables S52–S54), and the XII subgroup was mainly involved in pathogen recognition [39]. The proportion of XII RLKs and non-XII RLKs mirrored that of prRLPs and bRLPs, respectively (Fig. 4C). We found that *NLR* genes in *T. glandulifera* experienced a high proportion of gene loss after WGD, whereas the *NLR* duplicates were more retained in *L. japonica* (Supplementary Data Fig. S30). In *T. glandulifera*, the average expression levels of CNL (*P* = 0.0013) and TNL (*P* = 0.0003) *NLR* genes were significantly lower than those of *RNL *genes, as well as XII *RLK*s vs non-XII *RLK*s (*P* = 0.0240); the gene expression levels of *prRLP*s vs *bRLP*s were similar (Supplementary Data Fig. S31).

#### Convergent evolution of fast-evolving or positively selected genes in alpine plants

**Figure 5:**
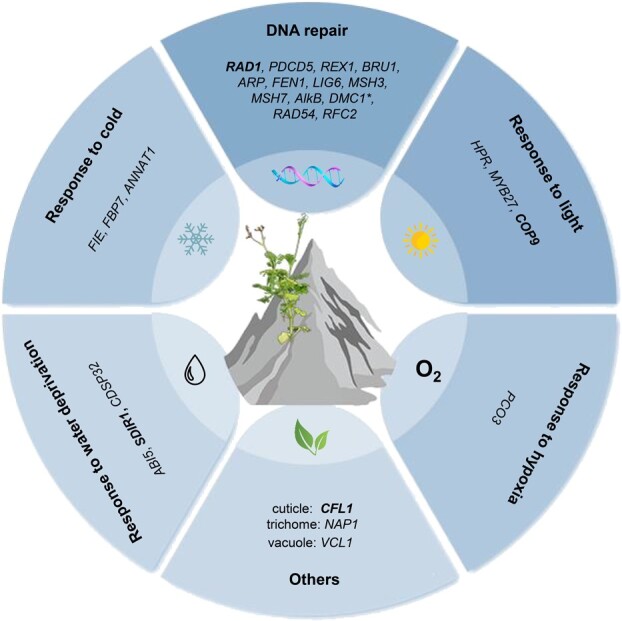
Fast-evolving or positively selected genes with potential functions for alpine adaptation in *T. glandulifera* and other four alpine plants. The gene labeled with an asterisk is an PSG and others are FEGs. The genes in bold black exhibited higher expression levels in *T. glandulifera* than its lowland relative *L. japonica*.

Convergent adaptation is often associated with genes under positive selection (PSGs) [40]. Similarly, genes with accelerated evolutionary rates (FEGs) are often associated with positive selection [41]. To identify genes that might have evolved due to specific adaptations of high-elevation plants, we detected FEGs with a significantly higher *K*_a_/*K*_s_ ratio in the high-elevation plants compared with the low-elevation ones and PSGs with a few sites under positive selection in high-elevation species (see Materials and methods section, Fig. 5, and Supplementary Data Table S55).

We identified 290 FEGs and six PSGs in the investigated alpine plants, and there was no overlap of FEGs and PSGs (Supplementary Data Table S55). Using the functional annotation information from the best BLAST hits of *A. thaliana* through UniPort and AceView, we found that 12 FEGs and one PSG were involved in DNA repair (e.g. *DMC1*, *RAD1*, *ARP*); four FEGs were involved in the response to cold (e.g. *FIE*, *FBP7*, *ANNAT1*); one FEG had a role in the response to light (e.g. *HPR*, *MYB27*, *COP9*); two FEGs were involved in the response to hypoxia (*PCO3*); and seven FEGs had a role in the response to water deprivation (e.g. *ABI5*, *SDIR1*, *CDSP32*) (Fig. 5, Supplementary Data Table S55). A series of genes associated with other stress responses as well as plant development were also found here, such as *CFL1* for cuticle development [42], *NAP1* for trichome morphogenesis [43], and *VCL1* for vacuole formation [44] (Fig. 5, Supplementary Data Table S55). In addition, eight genes were found to be upregulated in the alpine plant *T. glandulifera* compared with its lowland relative *L. japonica*, such as *RAD1* (TgChr02G08093.1), *SDIR1* (TgChr05G20036.1), *COP9* (TgChr01G01573.1), and *CFL1* (TgChr08G26115.1) (Supplementary Data Table S18).

## Discussion

In response to selective pressures posed by the alpine environment (e.g. low temperature, low oxygen, high UV-B radiation), high-elevation plants have evolved a series of adaptations [3, 4]. Here, we generated a chromosome-level genome assembly of the high-elevation species *T. glandulifera* (Caprifoliaceae) (Fig. 1B), and performed comparative genomic/transcriptomic analyses with its low-elevation relative *L. japonica*; we also included high- vs low-elevation comparisons of other phylogenetically distant alpine plants, to explore the potential genetic basis for the alpine adaptations.

Our genomic analysis revealed one relatively recent WGD event in the *T. glandulifera* genome, which was estimated to occur just before the diversification of Dipsacales during the Late Cretaceous (Fig. 2A). The preferential gene retention after WGD increased the copy number of cold-related genes in *T. glandulifera*, especially the positive regulators in the CBF-dependent cold signaling pathway (Supplementary Data Figs S10, S12, S15, S17, and S19). This finding highlights the potentially important role of WGD events for plants to colonize cold environments, such as high elevations or high latitudes [45–48].

By comparing differences in gene expression between *T. glandulifera* and *L. japonica* (Fig. 3E), we found that several highly expressed genes in *T. glandulifera* have potential functions for high-elevation adaptation, such as *CBF*s and *LEA*s for cold tolerance, *ERF-VII*s and *ADH1*s for hypoxia response, and *RAD1* and *RAD51C* for DNA repair [23–28] (Supplementary Data Table S18). This implies that increasing the expression levels of some important genes is also an important adaptation strategy in alpine plants.

Through comparative genomic analysis among five pairs of high- vs low-elevation species, including *T. glandulifera* and *L. japonica*, we revealed that the gene families related to disease resistance have been contracted in a convergent fashion across five alpine plants compared with their lowland relatives (Fig. 4). Here, not only *NLR*s, as found in the previous study [5], but also two other key components of the plant immune receptors (*RLP*s and *RLK*s families) were observed to be reduced in the gene repertory size in alpine plants (Fig. 4). The observed streamlining of the alpine plant immune system is likely due to the relaxed selection pressure from pathogens, as microbial richness and diversity are lower in alpine regions than in the corresponding lowlands [49, 50]. Similarly, contraction of plant immune receptors has also been found in aquatic, parasitic, and carnivorous plants, where reduced pressure from pathogens is evident [51]. The loss of genes related to immune response was also reported in some animals, and has similarly been suggested to be another adaptation to alpine living [52, 53].

The loss of immunity genes makes sense energetically. For example, the immunity conferred by NLRs requires extensive energy, given that the activation of NLRs requires ATP binding [54]. In addition, disease resistance responses (PTI and/or ETI) could negatively regulate the expression of development-related genes [55]. Therefore, unnecessary immune induction or a high copy number of genes used for disease resistance when there is limited challenge from pathogens would be selected against [56]. In alpine environments, low oxygen dramatically reduces the efficiency of cellular ATP production [57], and low temperature also inhibits the rate of ATP synthesis [58]. So, the co-contraction of the gene repertoire size for plant immune receptors in alpine plants reflects an energy-saving strategy for survival in hostile alpine environments [59]. In each family of plant immune receptors, the gene repertory size of the clades for pathogen recognition were found to exhibit a convergent reduction in alpine plants, such as sensor *NLR*s (*CNL*s) [34], *prRLP*s [38], and XII *RLK*s [39] (Fig. 4B and C), whereas helper *NLR*s (*RNL*s) for the transduction of immune signals [34], *bRLP*s and non-XII *RLK*s related to growth and development [38, 39] showed nearly no change in gene repertory size in high- vs low-elevation species (Fig. 4B and C). As a whole, the genes for pathogen recognition showed relatively lower expression levels than the ones for survival and growth in the same gene family (Supplementary Data Fig. S31). This fits the idea that immune receptors would be perfectly ‘off’ in the absence of a trigger pathogen [60].

In addition to the molecular convergence in gene copy number, we also found a group of genes with a convergent change of gene evolutionary rate in the investigated high-elevation plants (Supplementary Data Table S55). The putative functions of these genes included DNA repair, response to cold, and response to hypoxia, etc. (Fig. 5, Supplementary Data Table S55), which are all related to the major environmental stresses inhibiting the survival and growth of alpine plants. Three genes, *HPR*, *MYB27*, and *ARP*, were also found to be PSGs in other studies of alpine adaptation [5, 61]. Strong UV radiation in alpine environments causes indirect damage to DNA. Here, 12 FEGs and 1 PSG for DNA repair were detected in the alpine plants, of which potential functions cover various modes of DNA repair (Fig. 5): DNA damage response (*RAD1*, *PDCD5*, *REX1*, and *BRU1*), base excision repair (*ARP*, *FEN1*, and *LIG6*), mismatch repair (*MSH3* and *MSH7*), direct reversal of damage (*AlkB*), homologous recombination repair (*DMC1* and *RAD54*), and nucleotide excision repair (*RFC2*) [27, 28]. These will be good candidates for future studies of gene function, to better understand the adaptation to DNA damage in alpine plants.

All of these findings suggest that alpine plants, including *T. glandulifera*, have developed diverse strategies to adapt to alpine habitats. Our study provides valuable genomic resources and important candidate genes for further in-depth exploration of high-elevation adaptation combining multi-omics data with an evolutionary developmental approach. Importantly, cold-adapted alpine plants with their streamlined immune systems are facing dual pressures from elevated temperatures as well as increased pathogen activity as a result of global climate change. We propose that conservation efforts for *T. glandulifera* and other alpine plants must prioritize the development of *in situ* and *ex situ* protection strategies that consider both biotic and abiotic factors.

## Materials and methods

### Plant materials, chromosome counting, and flow cytometric estimation

Plant materials of *T. glandulifera* (2*n* = 18) were collected from the Latimojong Ranges (altitude 3132 m, 120° 02′ 72″ E, 3° 39′ 47″ N) in Sulawesi Selatan of Indonesia (Fig. 1A). The voucher specimen (no. CPG33179) was stored at the National Plant Specimen Resource Center. Tissue samples (fresh leaves, stems, roots, and flowers) were collected and preserved at −80°C for DNA/RNA extraction and sequencing library preparation. To generate transcriptome data representing Viburnaceae and all subfamilies in Caprifoliaceae, fresh leaves from 13 other species of Dipsacales were collected and stored in RNAlater™ stabilization solution (Invitrogen, CA, USA) at −80°C: samples of *Patrinia scabiosifolia* and *Scabiosa tschiliensis* from Baihua Mountain, Beijing, China; samples of *Morina kokonorica* from Nangqian County, Yushu Tibetan Autonomous Prefecture, Qinghai, China; and all the other samples (*Dipsacus fullonum*, *Valeriana officinalis*, *Dipelta floribunda*, *Kolkwitzia amabilis*, *Abelia chinensis*, *Zabelia biflora*, *Symphoricarpos orbiculatus*, *Weigela coraeensis*, *Sambucus williamsii*, and *Viburnum opulus*) from the National Botanical Garden, Beijing, China (Supplementary Data Table S13).

Chromosomes of *T. glandulifera* during root-tip mitosis were visualized using a DM6B fluorescence microscope (Leica Corporation, Wetzlar, Germany) (Fig. 1A). Fresh leaves of *T. glandulifera* were sampled for flow cytometric assays with three technical replicates after propidium iodide staining using a BD LSRFortessa™ cell analyzer (BD Biosciences, NJ, USA) (Supplementary Data Fig. S2). We used *Oryza sativa* ssp. *japonica* [62] as the reference standard.

### Sequencing and assembly

We used a combination of sequencing technologies: Illumina, PacBio, 10x Genomics, and Hi-C (high-throughput chromosome conformation capture) for the sequencing and assembly of the *T. glandulifera* genome based on the NovaSeq 6000 platform (Illumina, CA, USA) and PacBio Sequel platform (Pacific Biosciences, CA, USA) (Supplementary Data Table S1). The steps of library construction and sequencing were performed at Novogene (Beijing, China). To obtain enough corrected reads for *de novo* assembly, the longest subreads were initially selected as seed reads to correct sequence errors in the PacBio sequencing. *De novo* assembly using the corrected reads to produce primary contigs was performed by FALCON (v.0.3.0) [63]. The primary contigs were then polished using Quiver (v.2.3.3) [64] based on corrected PacBio long reads and using Pilon (v.1.18) [65] based on clean Illumina short reads. The total length of the assembly version 0.1 (V0.1) was 698.97 Mb with a contig N50 size of 1.87 Mb (Supplementary Data Table S2). Then, we used BWA-MEM (0.7.12-r1039) [66] to align the 10x Genomics data to the primary assembly and used FragScaff (v.2.1) [67] to extend the contigs into initial scaffolds. The scaffold N50 size reached 3.78 Mb in this new assembly (V0.2) (Supplementary Data Table S2). Subsequently, the Hi-C sequence data were aligned to the assembled scaffolds by BWA-MEM (0.7.12-r1039) [66], and the scaffolds were clustered into chromosomes with Lachesis (v.2.1) [68]. The final assembly (V0.3) was 680.38 Mb with a scaffold N50 size of 66.68 Mb and >91.25% in nine pseudomolecules (Supplementary Data Fig. S3, Supplementary Data Tables S2–S4).

RNA-sequencing (RNA-seq) libraries were prepared at Novogene (Beijing, China) and sequenced on the Illumina NovaSeq 6000 platform. *Triplostegia glandulifera* RNA-seq data were used for genome evaluation, gene annotation, and RNA-seq differential expression analysis (Supplementary Data Tables S7, S10, and S17). Transcriptomes were assembled from filtered reads using Trinity (v.2.11.0) [69]. In addition, we obtained transcriptome data for 13 species of Dipsacales as mentioned above (Supplementary Data Table S13) through RNA-seq. We used published RNA-seq data for two species, *Nardostachys jatamansi* (SRX7804715) [70] and *Diabelia spathulata* (DRX054302) [71]; they were also included in the transcriptome assembly (Supplementary Data Fig. S4, Supplementary Data Table S13).

### Genome repeats and gene annotation

We identified tandem repeats using Tandem Repeats Finder (TRF) (v.4.09) [72] (Supplementary Data Table S8). We predicted repetitive elements in the genome using RepeatMasker (v.4.0.7) and Repeat ProteinMask (v.4.0.7) [73] approaches using a repeat library from the RepBase [74] (Supplementary Data Table S8 and S9). The repeat-masked genome was used for gene annotation. For *de novo* gene prediction, we utilized several tools, including AUGUSTUS (v.3.0.3) [75], GlimmerHMM (v.3.0.4) [76], SNAP (2013-11-29) [77], GeneID (v.1.4) [78], and Genescan (v.1.0) [79] (Supplementary Data Table S10). For homology-based gene prediction, the protein evidence was drawn from orthologous proteins of *Arabidopsis. thaliana*, *D. carota*, *Helianthus annuus*, *Ipomoea nil*, *Olea europaea*, and *Solanum lycopersicum* (Supplementary Data Table S10).

For RNA-seq-assisted gene prediction, Cufflinks (v.2.2.1) [80] and PASA (Program to Assemble Spliced Alignment) (v.2.4.1) [81] tools were used (Supplementary Data Table S10). The non-redundant reference gene set was generated by merging gene models predicted by the above methods with EVM (EVidenceModeler) (v.1.1.1) [82] and then updated by PASA (v.2.4.1) [81] (Supplementary Data Table S10). Functional annotation of predicted protein-coding genes was based on BLAST results from the Swiss-Prot, TrEMBL, Pfam, InterPro, and EggNOG databases (Supplementary Data Table S11). Non-coding RNAs include miRNAs, tRNAs, rRNAs, and snRNAs (Supplementary Data Table S15). To annotate miRNAs and snRNAs, we searched the Rfam database (13.0) [83] using Infernal (v.1.1.2) [84]. To identify tRNAs, we employed tRNAscan-SE (v.1.3.1) [85], and rRNAs were predicted by aligning the *A. thaliana* rRNA sequences against the *T. glandulifera* genome using BLASTN (E-value cut-off of 1 × 10^−5^).

### Phylogenomic analysis and divergence time estimation

The genomes or transcriptomes of *T. glandulifera* and 14 Caprifoliaceae species (*D. fullonum*, *S. tschiliensis*, *V. officinalis*, *N. jatamansi*, *P. scabiosifolia*, *D. floribunda*, *K. amabilis*, *A. chinensis*, *D. spathulata*, *Z. biflora*, *Morina kokonorica*, *L. japonica*, *S. orbiculatus*, and *Weigela coraeensis*), two Viburnaceae species (*S. williamsii* and *V. opulus*), and one species of Apiaceae species (*D. carota*) were used for gene family clustering by OrthoFinder (v.2.3.3) [86] with default parameters (Supplementary Data Table S14). The longest transcript for each gene locus was selected as the representative transcript of the gene for further gene clustering analysis.

We used 106 common single-copy genes identified by OrthoFinder (v.2.3.3) [86] for phylogeny reconstruction, and *D. carota* was used as an outgroup in the analysis. For each gene, amino acid sequences were aligned by MAFFT (v.7.310) [87], and then nucleotide sequences were aligned according to the corresponding amino acid alignments using PAL2NAL (v.14) [88]. We constructed phylogenetic trees using both a concatenated-based analysis and a coalescent-based approach (Supplementary Data Fig. S5). For the concatenated-based analysis, alignments were concatenated as single supermatrices and trees were inferred based on the PROTGAMMAAUTO and GTRGAMMA models of amino acid and nucleotide substitution, respectively, using RAxML (v.8.1.17) [89] with 1000 bootstrap replicates (Supplementary Data Fig. S5). For the coalescent-based approach, the phylogenetic tree of each single-copy gene was further constructed to infer a consensus species tree using ASTRAL (v.5.5.11) [90] with quartet scores and posterior probabilities (Supplementary Data Fig. S5).

To estimate divergence times, we applied MCMCTree in the PAML package (v.4.7) [91] with three calibration constraints. These included a secondary calibration at the stem node of Dipsacales, which ranged from 80.15 to 98.62 Mya [92]; a fossil constraint for the most recent common ancestor (MRCA) of *D. floribunda* and *K. amabilis*, which we assigned to 48–56 Mya [93], and a fossil constraint for the MRCA of *Viburnum* and *Sambucus* which we assigned to 45–86 Mya [94] (Fig. 2A).

### Identification of whole-genome duplications

We applied the wgd (1.0.1) tool [95] to construct *K*_s_ distributions among all paralogs (whole paranome) from the 17 Dipsacales species as mentioned above and one Apiaceae (*D. carota*) (Fig. 2C, Supplementary Data Fig. S6). The paralogs from three genomes (*T. glandulifera*, *L. japonica*, and *D. carota*) were filtered according to the co-linearity analysis by i-ADHORE (3.0) [96]. Based on the fitted mixture model (BGMM) in wgd (1.0.1) [95], we fitted the *K*_s_ distribution of paralogs from each hypothesized WGD peak and estimated the mean and variance of each WGD peak (Fig. 2C, Supplementary Data Fig. S6). To distinguish *K*_s_-based age distributions between WGD and speciation, we further identified the ortholog *K*_s_ distributions between *T. glandulifera* and *P. scabiosifolia*, *W. coraeensis*, *S. williamsii*, and *D. carota* (Fig. 2C). The ages of WGDs and ortholog divergence were inferred by the formula *T* = *K*_s_/2*r*, and the same *r* value was used to calculate the time of WGD events for each species (Fig. 2A, Supplementary Data Table S16.

As a second approach, we used Multi-tAxon Paleopolyploidy Search (MAPS) [97] to confirm the placement of ancient WGDs (Fig. 2B, Supplementary Data Table S15). Based on the hypothesized WGD peaks in Dipsacales, eight species (*D. fullonum*, *S. tschiliensis*, *T. glandulifera*, *K. amabilis*, *L. japonica*, *W. coraeensis*, *S. williamsii*, and *D. carota*) were selected for gene family clustering (Fig. 2B). Both null and positive simulations of the background gene birth and death rates were performed to compare with the observed number of duplications at each node (Fig. 2B). Third, we investigated synteny among *T. glandulifera*, *L. japonica*, and *V. vinifera* using MCScan (Python version) [98] and used the syntenic depth function in MCScan to estimate duplication history in the respective genomes (Fig. 2D, Supplementary Data Figs S7 and S8).

### Gene expression and functional enrichment analyses

We utilized Salmon [99] to quantify transcript abundance across replicate samples based on TPM (transcripts per million) from RNA-seq reads of *T. glandulifera* and *L. japonica* leaves [100] (Supplementary Data Table S17). The subsequent gene differential expression analysis was based on one-to-one orthologous genes between *T. glandulifera* and *L. japonica* through a best reciprocal BLAST (E-value cut off <1 × 10^−10^), and was performed using the R package edgeR [101] (Fig. 3E). We obtained adjusted *P*-values to account for the false discovery rate (FDR) in edgeR [101]. The |log_2_(Fold Change)| >2 and FDR <0.01 were set as the threshold to identify significant DEGs. The GO (Gene Ontology) and KEGG (Kyoto Encyclopedia of Genes and Genomes) functional enrichment analyses of DEGs were implemented by the R package clusterProfiler (v.3.6.0) [102] (*P*-value <0.05, *q*-value <0.05) (Fig. 3E, Supplementary Data Table S18).

### Gene family expansion and contraction

We measured the expansion and contraction of gene families in the *T. glandulifera* genome by comparing it with the available genomes of 10 other angiosperm species from closely related genera and families (*L. japonica*, *D. carota*, *H. annuus*, *S. lycopersicum*, *I. nil*, *O. europaea*, *A. thaliana*, *Gossypium raimondii*, *O. sativa*, and *Sorghum bicolor*) using CAFÉ (v.4.2) [29] (Supplementary Data Fig. S23, Supplementary Data Tables S19 and S20). We further investigated significant expansion or contraction of gene families in high-elevation plants (*T. glandulifera*, *Erigeron breviscapus*, *Rhododendron williamsianum*, *Crucihimalaya himalaica*, and *Salix brachista*) relative to their relatives from low elevations (*L. japonica*, *Erigeron canadensis*, *Rhododendron ovatum*, *Capsella rubella*, and *Salix viminalis*) (Supplementary Data Table S21) by comparing the total number of genes per orthogroup between the two types using a binomial test (Fig. 4A). We retained orthogroups showing a statistically significant outcome in terms of gene counts (adjusted *P*-value <0.05) (Supplementary Data Table S40).

### Identification of cold-related genes and plant immune receptor genes

In *T. glandulifera* and other species of Dipsacales, the homologs of well-known cold response regulators (*CBF*s, *COLD1*, *CRLK1/2*, *RGA1/2*, *OST1*, *SIZ1*, *ICE1/2*, *CAMTA*s, *CCA1/LHY*, *BES1/BZR1*, and *CESTA*) in the CBF-dependent cold signaling pathway [23, 24] were initially identified by BLASTP with an E-value cut-off of 1 × 10^−10^ using the related genes from *A. thaliana* as queries, and were further confirmed using phylogenetic analyses (Fig. 3A, Supplementary Data Figs S12–S22). We also surveyed the main source of cold-responsive genes, LEA proteins, in *T. glandulifera*, *L. japonica*, *D. carota*, and *A. thaliana* using HMMER (v.3.3.2) [103] based on eight Pfams: Dehydrin (DHN) (PF00257), LEA_1 (PF03760), LEA_2 (PF03168), LEA_3 (PF03242), LEA_4 (PF02987), LEA_5 (PF00477), LEA_6 (PF10714), and Seed Maturation Protein (SMP) (PF04927) [104] (Fig. 3B and Supplementary Data Table S33). The well-known *Arabidopsis* LEAs were also used in the phylogenetic analysis [105] (Fig. 3B).

In the above-mentioned five pairs of high- vs low-elevation plants, three types of plant immune receptor genes (NLRs, RLKs, and RLPs) were identified (Supplementary Data Tables S46–S54). The related canonical genes from *A. thaliana* were used as references (Supplementary Data Tables S41, S43, and S45). The canonical NLR sequences were screened using HMMER (v.3.3.2) [103] to search for the NB-ARC (Pfam: PF00931) domain [106] with an E-value cut-off of 1 × 10^−5^. Extracted sequences were further checked for protein domains (TIR, CC, and LRR) [107] to remove false-positive hits using Pfam (http://pfam.janelia.org/) and COILS [108]. Full-type NLRs with >160 amino acids were used for further analyses [109]. The canonical RLK sequences were identified using the *A. thaliana* RLKs [110] as queries through BLASTP searches (E-value cut-off <1 × 10^−10^). We also checked the LRR and kinase domains using Pfam (http://pfam.janelia.org/) and the TM domain using TMHMMv.2.0 (https://services.healthtech.dtu.dk/service.php?TMHMM-2.0) [111, 112]. Distinguished from RLKs, RLPs lack the kinase domain [113]. We used *A. thaliana* RLPs [114] as queries to BLASTP (E-value cut off <1 × 10^−10^) against the proteins of the 10 target species and only kept sequences with TM and LRR domains and without the kinase domain for further analyses. We also included the well-known functional NLRs and RLPs of 18 other species in the phylogenetic analyses (Supplementary Data Tables S42 and S44).

Amino acid sequence alignments were performed in MAFFT (v.7.310) [87] with default parameters. Maximum likelihood trees were built using IQTREE (v.2.0) [115] with 1000 replicates of ultrafast bootstrap.

### Identification of fast-evolving genes and positively selected genes

The one-to-one orthologs among the above-mentioned five pairs of high- vs low-elevation plants were extracted using OrthoFinder (v.2.3.3) [86]. Then each orthologous gene set was aligned using MAFFT (v.7.310) [87] and trimmed using GBlocks [116]. To detect the common FEGs of high-elevation species, we used the branch model in CODEML from PAML [91]. High-elevation species were set as the foreground branches and low-elevation species as background branches. The null model assumed that all branches evolved at the same rate, and the alternative model allowed the foreground branch to evolve under a different rate. To detect positive selection on a few codons along high-elevation species, we used the optimized branch-site model in CODEML from PAML [91], with one model allowing sites to be under positive selection on the foreground branch and another model assuming sites to evolve neutrally and under purifying selection. The likelihood ratio test was employed to determine significant differences between different models for each orthologs. The false discovery rate (FDR) in multiple testing was used to correct significance levels of *P*-values [117]. The genes with an FDR-adjusted *P*-value <0.05 and a higher dN/dS in the foreground branch than in the background branch were considered as FEGs. The genes with an FDR-adjusted *P*-value <0.05 and an ω >1 of the foreground branch were considered as PSGs.

## Supplementary Material

Web_Material_uhae077

## Data Availability

The raw sequencing data of the *T. glandulifera* genome and transcriptome have been deposited in the National Genomics Data Center (https://ngdc.cncb.ac.cn/, CNCB) under the BioProject accession number PRJCA019252. The *T. glandulifera* genome assembly and annotation files have been submitted to FigShare (http://doi.org/10.6084/m9.figshare.25018103). The RNA-seq reads of 13 Dipsacales species are available from CNCB under the BioProject accession number PRJCA019358. Public transcriptomes used in this study are available from NCBI under the accession numbers SRX7804715 and DRX054302. All data are available from the corresponding author upon reasonable request.
