# Supplementary material for: Coping with alpine habitats: genomic insights into the adaptation strategies of Triplostegia glandulifera (Caprifoliaceae)
Source: Hortic Res. 2024 May 1;11(5):uhae077. doi: 10.1093/hr/uhae077 (PMC11109519; doi:10.1093/hr/uhae077)
Supplement: Web_Material_uhae077 [file web_material_uhae077.zip › Supplemental Data Figure S2.pdf]

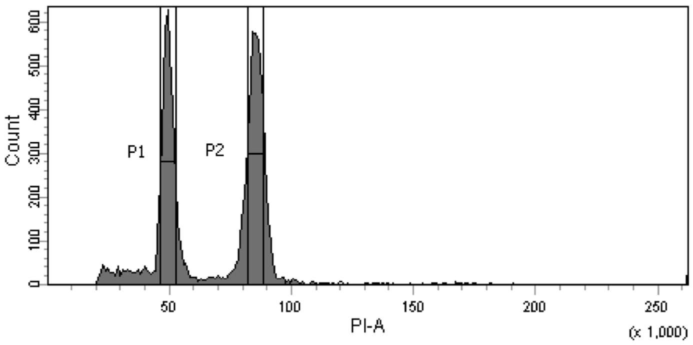

Number	*Oryza sativa*		*Triplostegia glandulifera*		Multiple relation	Estimated genome size (Mb)
	MFI (P1)	CV(%)	MFI (P2)	CV(%)	(P2/P1)	
1	48.99	3.3	85.06	2	1.74	669.62
2	46.26	3.8	79.87	2.1	1.73	666.02
3	47.58	3.6	82.47	2	1.73	668.5
mean±SD	47.61±1.37		82.47±2.59		1.73±0.00	668.05±1.84

MFI = median fluorescent intensity

Supplemental Data Figure S2. Genome size estimation by flow cytometry. Using *Oryza sativa* (385.7 Mb) as the reference standard**.**
