# Supplementary material for: Coping with alpine habitats: genomic insights into the adaptation strategies of Triplostegia glandulifera (Caprifoliaceae)
Source: Hortic Res. 2024 May 1;11(5):uhae077. doi: 10.1093/hr/uhae077 (PMC11109519; doi:10.1093/hr/uhae077)
Supplement: Web_Material_uhae077 [file web_material_uhae077.zip › Supplemental Data Figure S4.pdf]

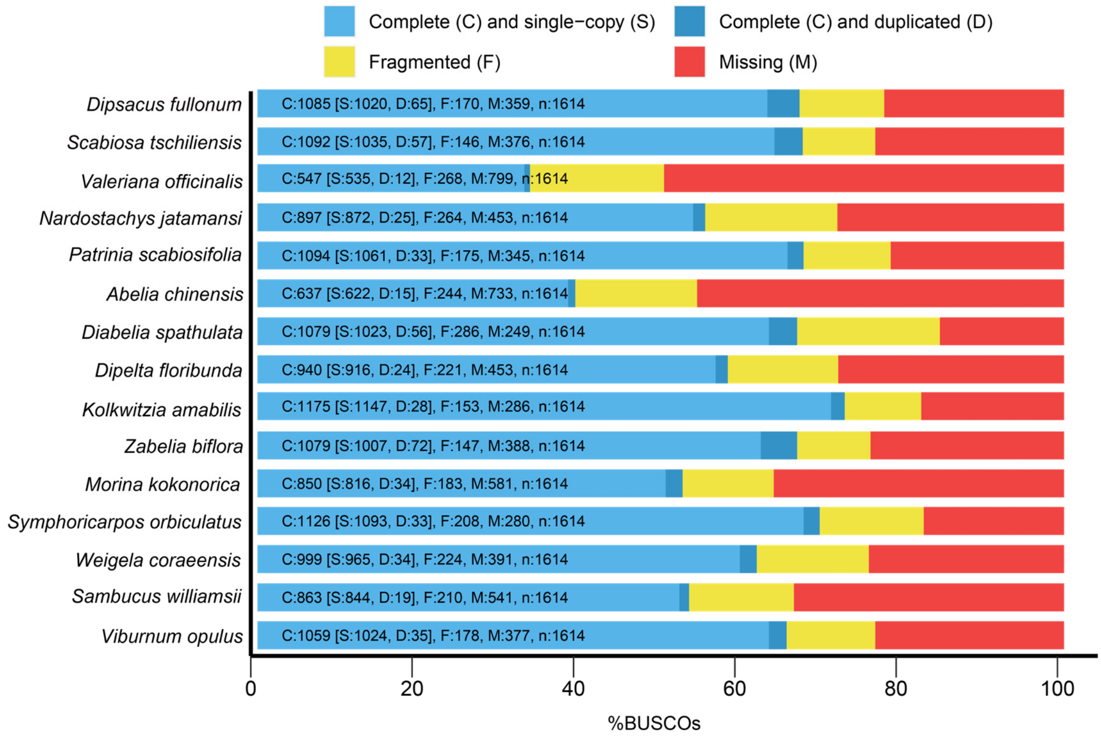

Supplemental Data Figure S4. BUSCO assessments of transcriptome assemblies. BUSCO scores of the transcriptome assemblies for 15 species in Dipsacales. The completeness of transcriptome was given as 'complete plus fragmented'.
