# Supplementary material for: Coping with alpine habitats: genomic insights into the adaptation strategies of Triplostegia glandulifera (Caprifoliaceae)
Source: Hortic Res. 2024 May 1;11(5):uhae077. doi: 10.1093/hr/uhae077 (PMC11109519; doi:10.1093/hr/uhae077)
Supplement: Web_Material_uhae077 [file web_material_uhae077.zip › Supplemental Data Figure S5.pdf]

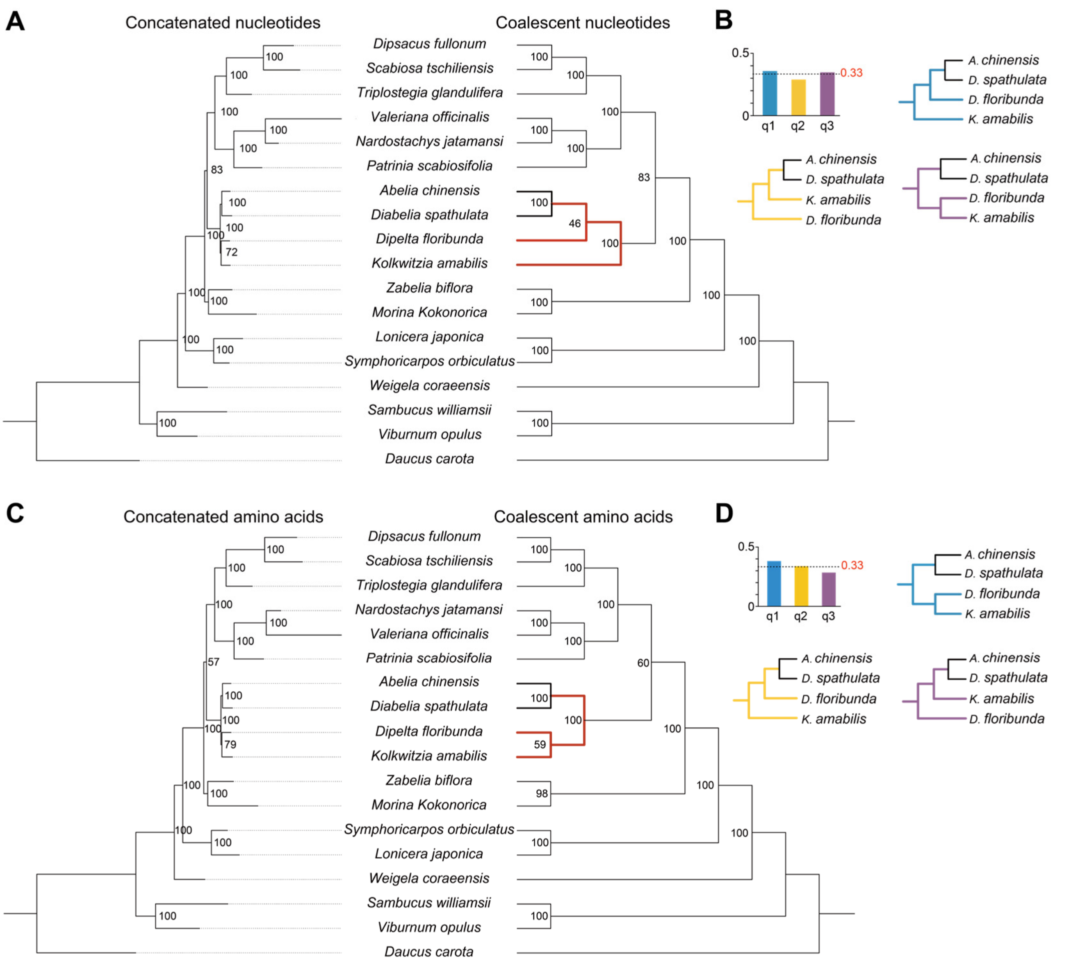

Supplemental Data Figure S5. Concatenated- and coalescent-based phylogenetic trees. **A** Phylogenetic trees based on the concatenated (left) (maximum likelihood (ML)) and coalescent methods (right) (ASTRAL) using nucleotide sequences. **B** Estimated proportions of the 106 single-copy gene trees based on nucleotide sequences. The x-axis labels q1, q2 and q3 indicate the quartet support for the three alternative topologies. The dashed line refers to a proportion of 0.33. **C** Phylogenetic trees based on the concatenated (left) and coalescent methods (right) using amino acid sequences. Interpretation is as in **A**. **D** Estimated proportions of the 106 single-copy gene trees based on amino acid sequences. Interpretation is same as **B**. Linnaeoideae with phylogenetic discordances are indicated. In most of our analyses, the topology of ((*Abelia chinensis*, *Diabelia spathulata*), (*Dipelta floribunda*, *Kolkwitzia amabilis*)) was strongly supported, with only slightly less supported Q value (the percentage of gene trees in support of the topology) than the topology of (((*Abelia chinensis*, *Diabelia spathulata*), *Dipelta floribunda*), *Kolkwitzia amabilis*) in the coalescent-based ASTRAL analysis of nucleotide sequences.
