# Supplementary material for: Coping with alpine habitats: genomic insights into the adaptation strategies of Triplostegia glandulifera (Caprifoliaceae)
Source: Hortic Res. 2024 May 1;11(5):uhae077. doi: 10.1093/hr/uhae077 (PMC11109519; doi:10.1093/hr/uhae077)
Supplement: Web_Material_uhae077 [file web_material_uhae077.zip › Supplemental Data Figure S6.pdf]

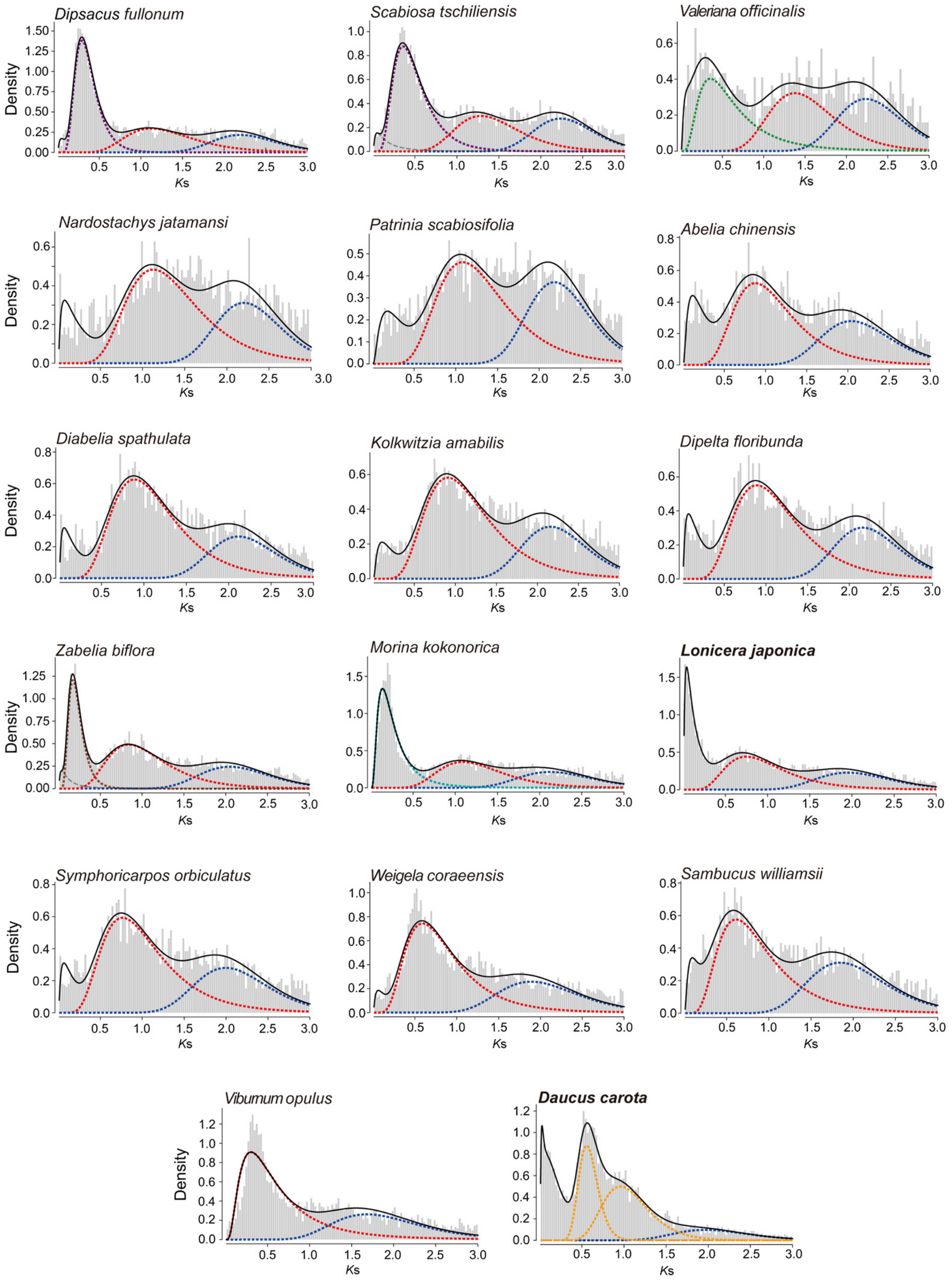

Supplemental Data Figure S6. *K*s distribution of the whole paranome for 16 Dipsacales species plus one outgroup (*Daucus carota*). *K*s distributions of paralogs are shown in grey and identified peaks are denoted by dotted lines. The names of species with sequenced genomes are in bold, and other data are from transcriptome sequencing. The red line shows D-WGD, blue line indicates γ-WGD, other lines present different lineage-specific or species-specific WGDs.
