# Supplementary material for: Coping with alpine habitats: genomic insights into the adaptation strategies of Triplostegia glandulifera (Caprifoliaceae)
Source: Hortic Res. 2024 May 1;11(5):uhae077. doi: 10.1093/hr/uhae077 (PMC11109519; doi:10.1093/hr/uhae077)
Supplement: Web_Material_uhae077 [file web_material_uhae077.zip › Supplemental Data Figure S7.pdf]

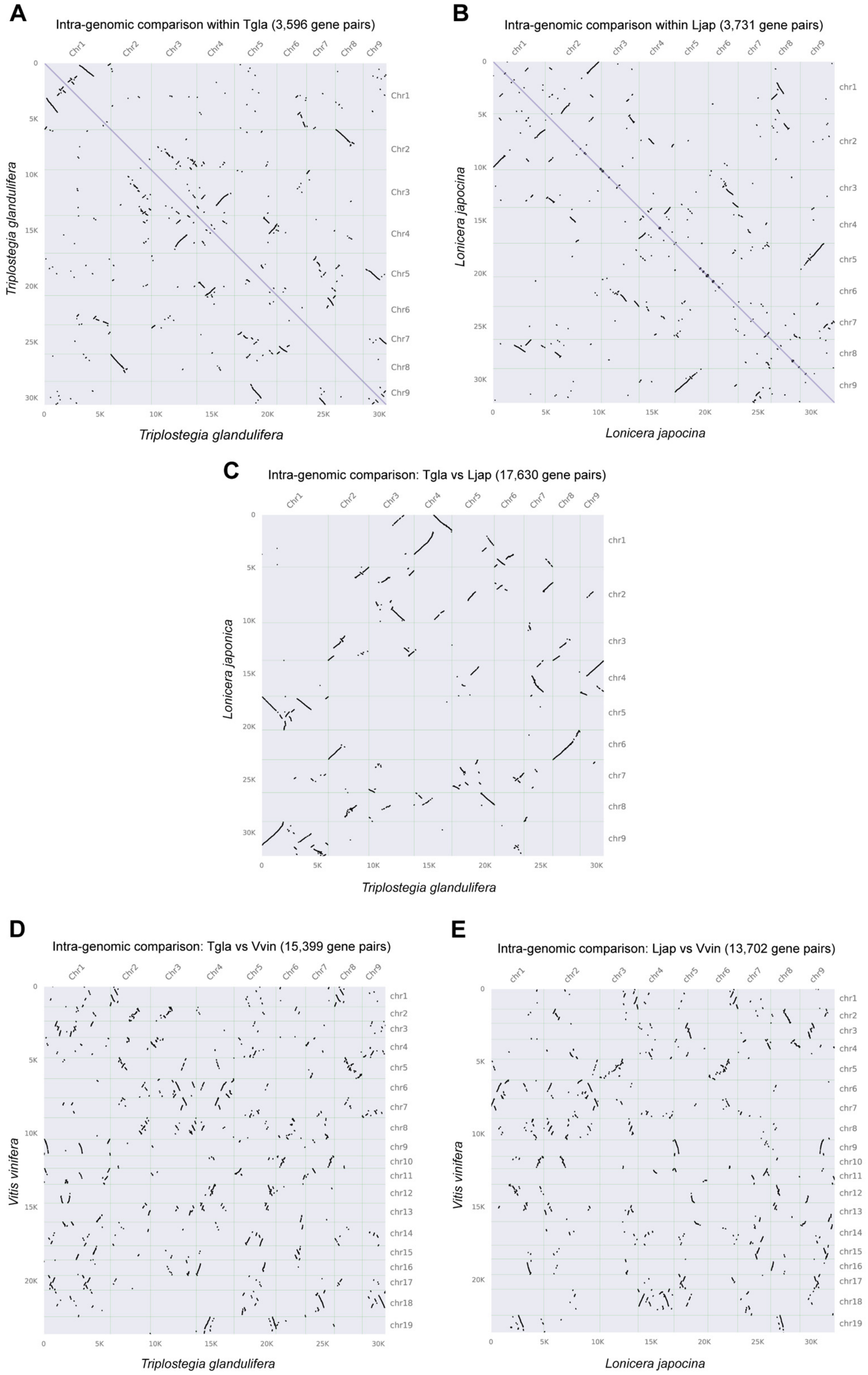

Supplemental Data Figure S7. Genomic comparison among *Triplostegia glandulifera*, *Lonicera japonica*, and *Vitis vinifera*. Synteny dotplot showing syntenic coding sequences between *T. glandulifera* and itself (**A**), *L. japonica* and itself (**B**), *T. glandulifera* and *L. japonica* (**C**), *T. glandulifera* and *V. vinifera* (**D**), *L. japonica* and *V. vinifera* (**E**).
