# Supplementary material for: Coping with alpine habitats: genomic insights into the adaptation strategies of Triplostegia glandulifera (Caprifoliaceae)
Source: Hortic Res. 2024 May 1;11(5):uhae077. doi: 10.1093/hr/uhae077 (PMC11109519; doi:10.1093/hr/uhae077)
Supplement: Web_Material_uhae077 [file web_material_uhae077.zip › Supplemental Data Figure S8.pdf]

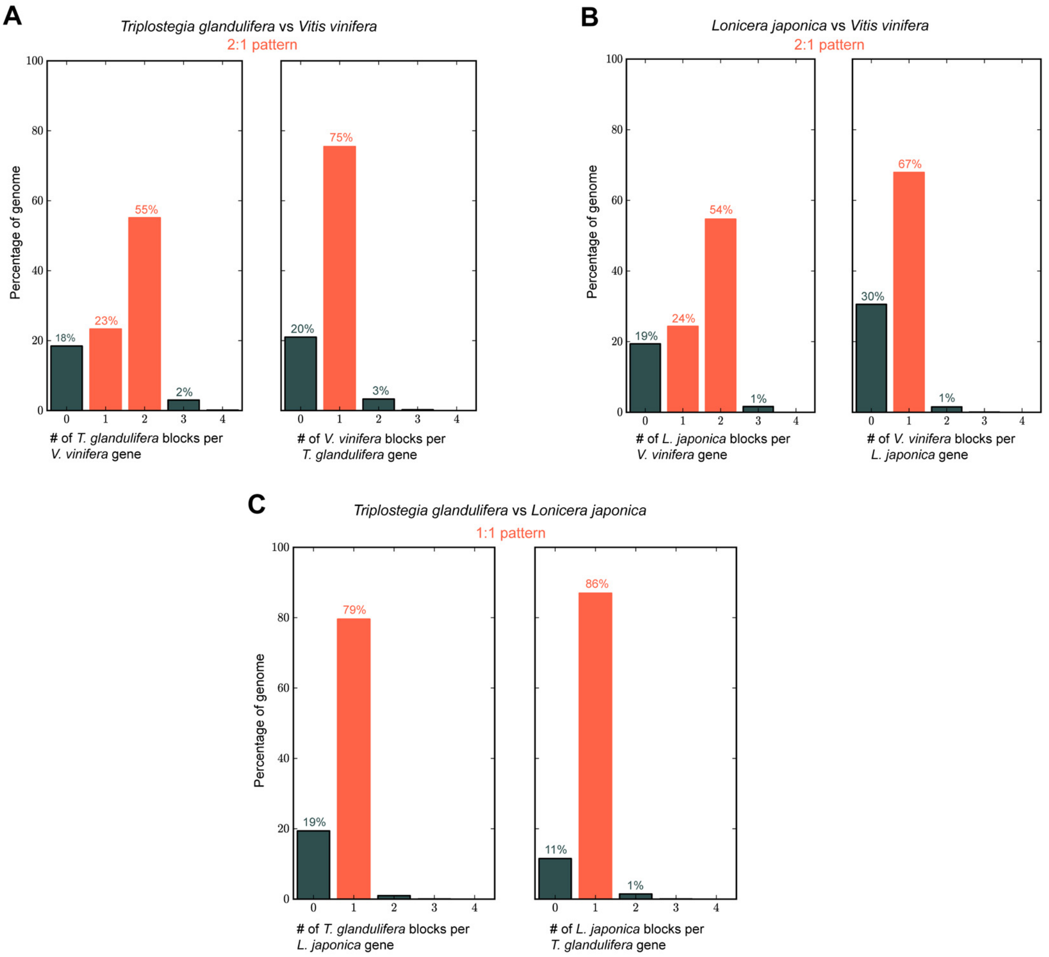

Supplemental Data Figure S8. The syntenic depth ratios among *Triplostegia glandulifera*, *Lonicera japonica*, and *Vitis vinifera*. Synteny depth ratio between *T. glandulifera* and *V. vinifera* was determined as 2:1 (**A**); *L. japonica* and *V. vinifera* as 2:1 (**B**); *T. glandulifera* and *L. japonica* as 1:1 (**C**).
