# Supplementary material for: Coping with alpine habitats: genomic insights into the adaptation strategies of Triplostegia glandulifera (Caprifoliaceae)
Source: Hortic Res. 2024 May 1;11(5):uhae077. doi: 10.1093/hr/uhae077 (PMC11109519; doi:10.1093/hr/uhae077)
Supplement: Web_Material_uhae077 [file web_material_uhae077.zip › Supplemental Data Figure S9.pdf]

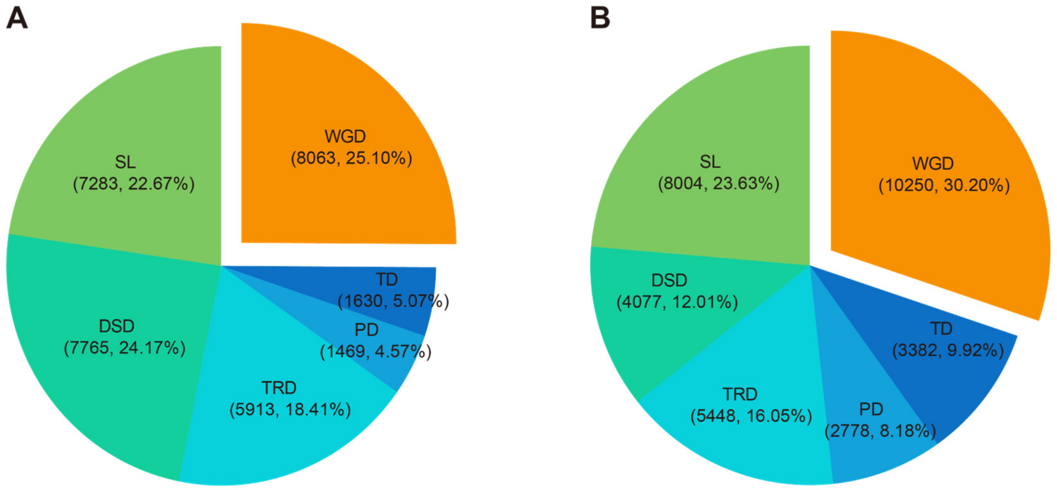

Supplemental Data Figure S9. The proportions of singletons and five modes of gene duplications in *Triplostegia glandulifera* (**A**) and *Lonicera japonica* (**B**). SL, singleton; WGD, whole-genome duplication; DSD, dispersed duplication; TRD, transposed duplication; TD, tandem duplication; PD, proximal duplication.
