# Supplementary material for: Coping with alpine habitats: genomic insights into the adaptation strategies of Triplostegia glandulifera (Caprifoliaceae)
Source: Hortic Res. 2024 May 1;11(5):uhae077. doi: 10.1093/hr/uhae077 (PMC11109519; doi:10.1093/hr/uhae077)
Supplement: Web_Material_uhae077 [file web_material_uhae077.zip › Supplemental Data Figure S11.pdf]

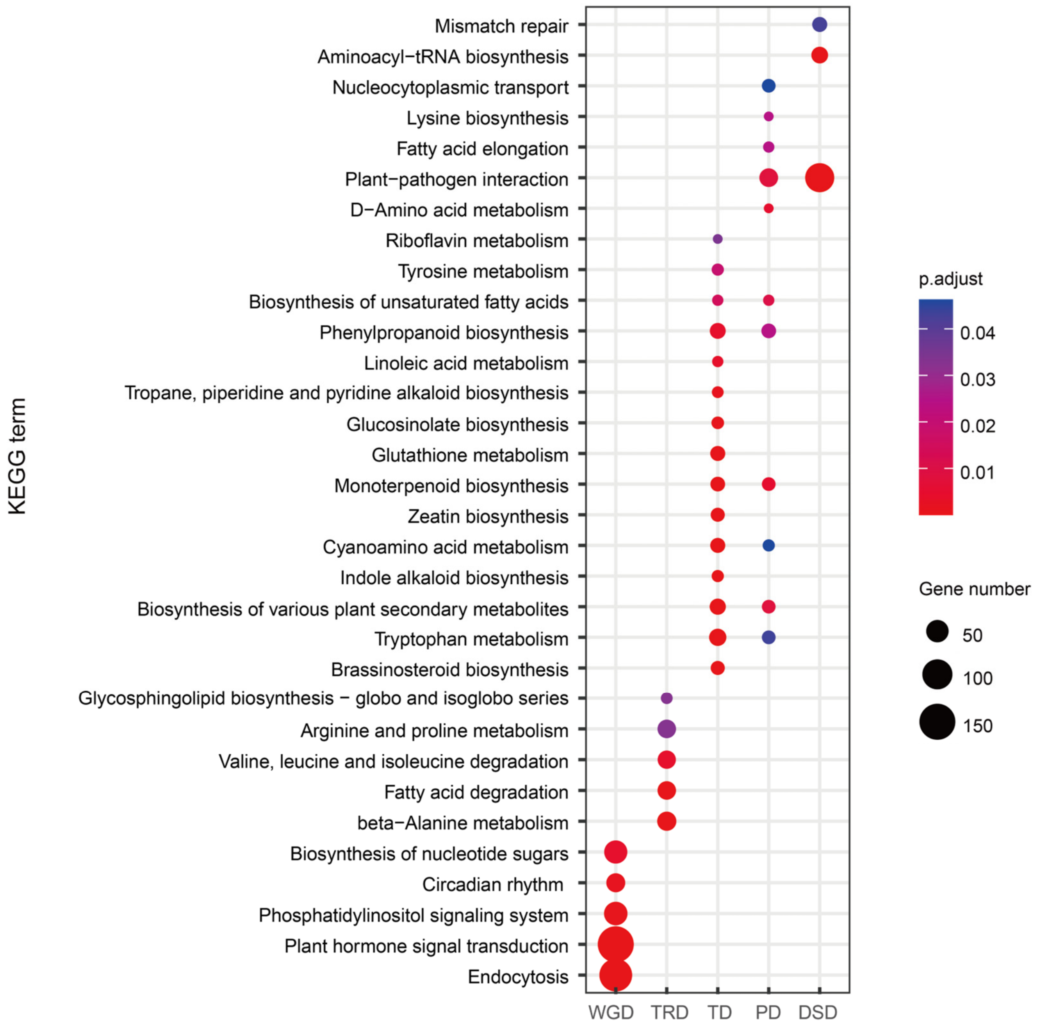

Supplemental Data Figure S11. The top 15 enriched KEGG pathways for genes derived from five modes of duplications (WGD, TRD, TD, PD, and DSD) in *Triplostegia glandulifera*. The color of circles displays the statistical significance of enriched KEGG pathways. The size of the circles shows the number of genes in a KEGG pathway. '*p*-adjust' is the adjusted *p*-value of the Benjamini–Hochberg false discovery rate (FDR).
