# Supplementary material for: Coping with alpine habitats: genomic insights into the adaptation strategies of Triplostegia glandulifera (Caprifoliaceae)
Source: Hortic Res. 2024 May 1;11(5):uhae077. doi: 10.1093/hr/uhae077 (PMC11109519; doi:10.1093/hr/uhae077)
Supplement: Web_Material_uhae077 [file web_material_uhae077.zip › Supplemental Data Figure S13.pdf]

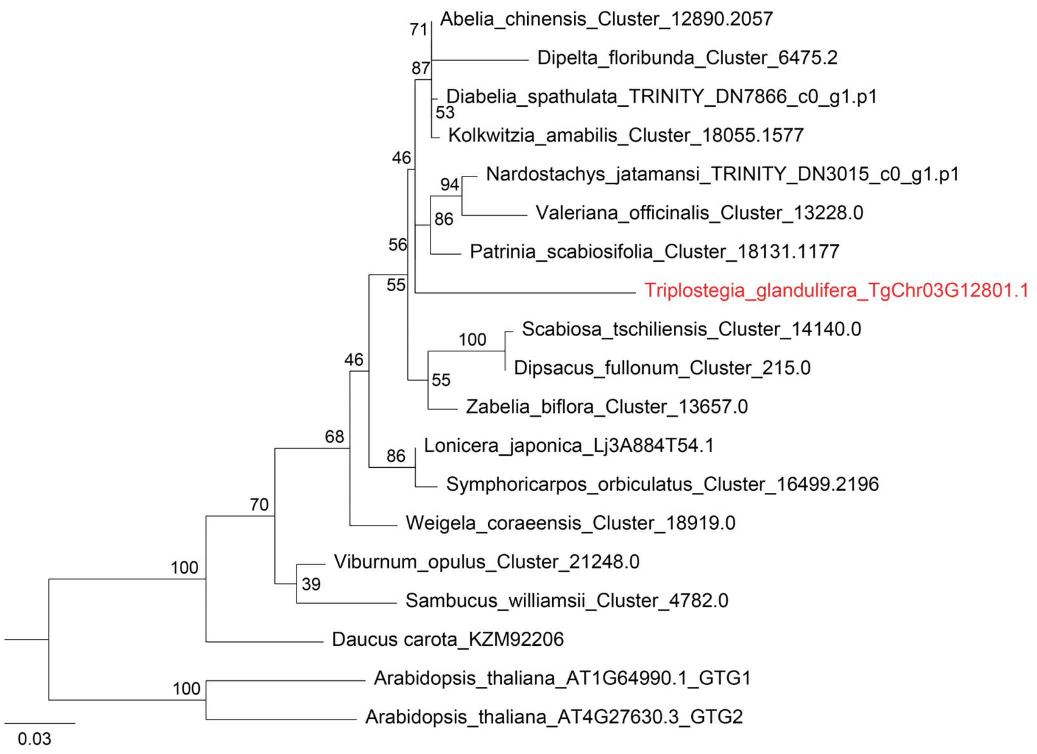

Supplemental Data Figure S13. Phylogenetic tree of *COLD1* (*GTG1/2*) genes in Dipsacales. The *GTG1/2* gene from *Daucus carota* is used as reference. The *GTG1/2* genes from *Arabidopsis thaliana* are used as outgroup and reference, which are the homologs of *COLD1* gene in *Oryza sativa*. Bootstrap support values were calculated using 1,000 replicates.
