# Supplementary material for: Coping with alpine habitats: genomic insights into the adaptation strategies of Triplostegia glandulifera (Caprifoliaceae)
Source: Hortic Res. 2024 May 1;11(5):uhae077. doi: 10.1093/hr/uhae077 (PMC11109519; doi:10.1093/hr/uhae077)
Supplement: Web_Material_uhae077 [file web_material_uhae077.zip › Supplemental Data Figure S22.pdf]

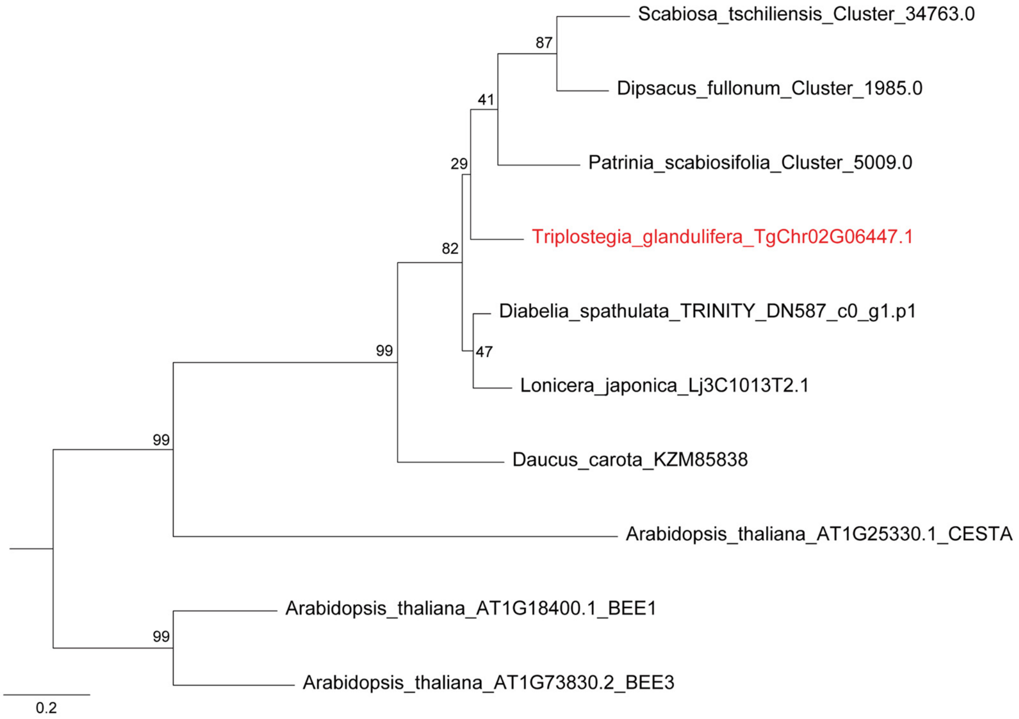

Supplemental Data Figure S22. Phylogenetic tree of *CESTA* genes in Dipsacales. The *CESTA* genes from *Daucus carota* and *Arabidopsis thaliana* are used as reference. The *BEE1/2* genes from *Arabidopsis thaliana* are used as outgroup. Bootstrap support values were calculated using 1,000 replicates.
