# Supplementary material for: Coping with alpine habitats: genomic insights into the adaptation strategies of Triplostegia glandulifera (Caprifoliaceae)
Source: Hortic Res. 2024 May 1;11(5):uhae077. doi: 10.1093/hr/uhae077 (PMC11109519; doi:10.1093/hr/uhae077)
Supplement: Web_Material_uhae077 [file web_material_uhae077.zip › Supplemental Data Figure S23.pdf]

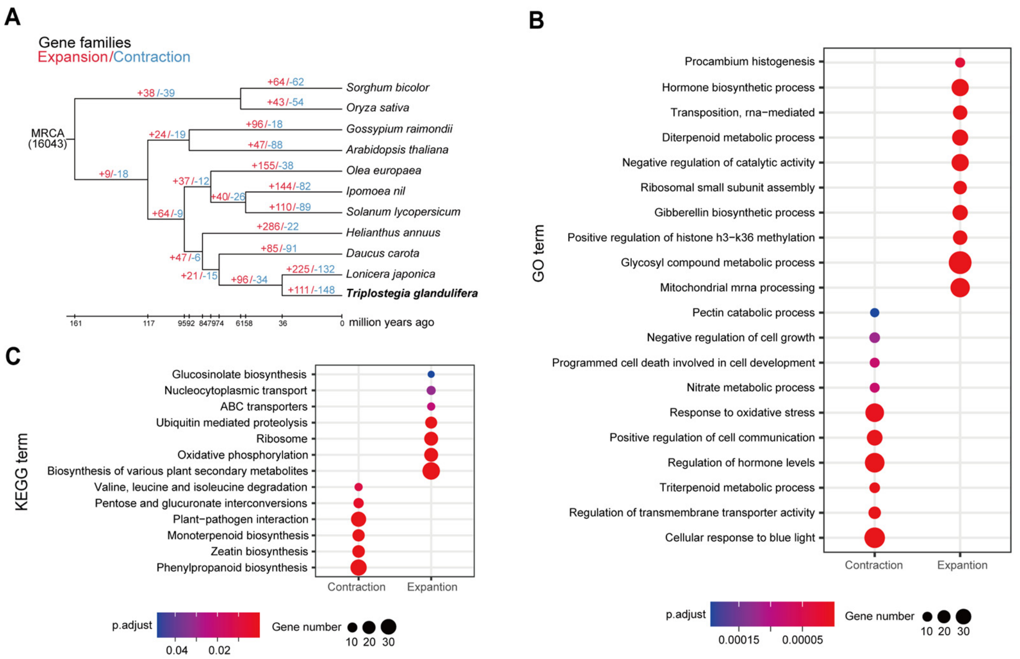

Supplemental Data Figure S23. Gene family contraction and expansion. **A** Numbers of contracted (blue) and expanded (red) gene families along the phylogenetic tree of *Triplostegia glandulifera* and other ten species. MRCA, most recent common ancestor. The tree was constructed on the basis of 306 single-copy gene families. **B** The top 10 enriched GO terms for contracted and expanded gene families in *T. glandulifera*. The size of the circles shows the number of genes in one GO term. The color of the circles displays the statistical significance of enriched GO terms. **C** KEGG enrichment analyses of contracted and expanded gene families in *T. glandulifera*. The size of the circles shows the number of genes in one KEGG pathway. The color of the circles displays the statistical significance of enriched KEGG pathways. '*p*-adjust' is the adjusted *p*-value of the Benjamini–Hochberg false discovery rate (FDR).
