# Supplementary material for: Coping with alpine habitats: genomic insights into the adaptation strategies of Triplostegia glandulifera (Caprifoliaceae)
Source: Hortic Res. 2024 May 1;11(5):uhae077. doi: 10.1093/hr/uhae077 (PMC11109519; doi:10.1093/hr/uhae077)
Supplement: Web_Material_uhae077 [file web_material_uhae077.zip › Supplemental Data Figure S24.pdf]

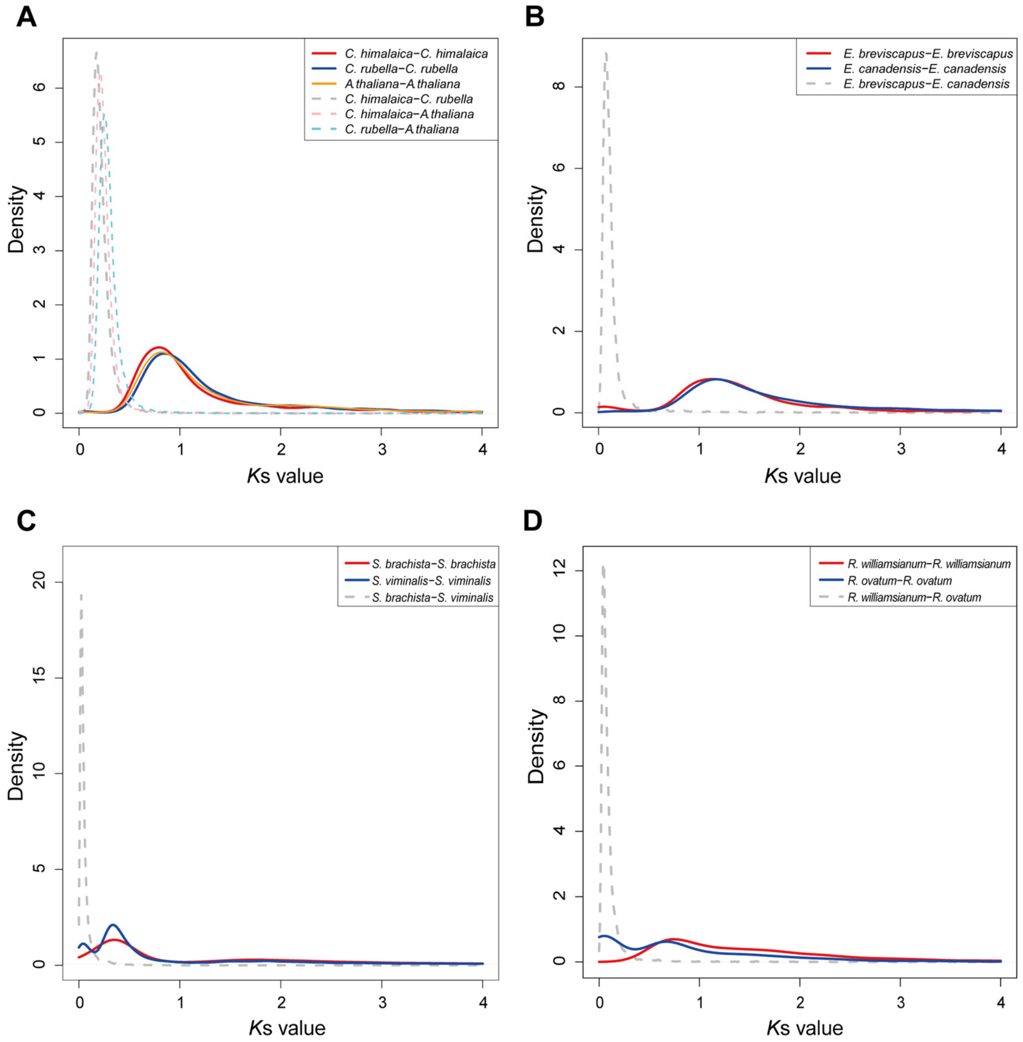

Supplemental Data Figure S24. Synonymous substitution rate (*K*s) density distributions of paralogs and orthologs of estimated species from four different families, respectively. **A** *Arabidopsis thaliana*, *Capsella rubella*, and *Crucihimalaya himalaica* from Brassicaceae. **B** *Erigeron canadensis* and *Erigeron breviscapus* from Asteraceae. **C** *Salix viminalis* and *Salix brachista* from Salicaceae. **D** *Rhododendron ovatum* and *Rhododendron williamsianum* from Ericaceae. The *K*s distributions show that there was one WGD event shared by corresponding estimated species in each family, but no species-specific WGD was found.
