# Supplementary material for: Coping with alpine habitats: genomic insights into the adaptation strategies of Triplostegia glandulifera (Caprifoliaceae)
Source: Hortic Res. 2024 May 1;11(5):uhae077. doi: 10.1093/hr/uhae077 (PMC11109519; doi:10.1093/hr/uhae077)
Supplement: Web_Material_uhae077 [file web_material_uhae077.zip › Supplemental Data Figure S25.pdf]

A

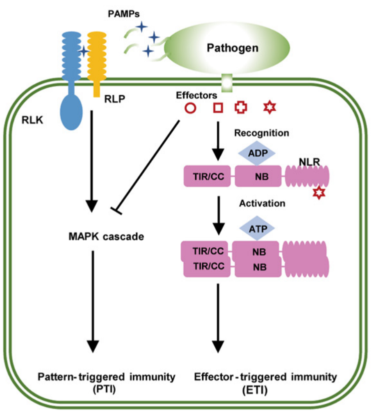

B

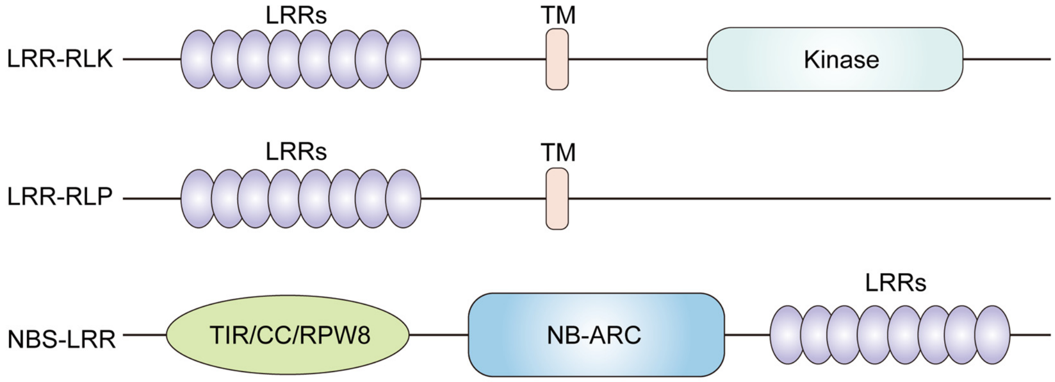

Supplemental Data Figure S25. Schematic diagram of plant immune systems (**A**) and schematic protein structure of LRR-RLKs, LRR-RLPs, NBS-LRRs (**B**).
