# Supplementary material for: Coping with alpine habitats: genomic insights into the adaptation strategies of Triplostegia glandulifera (Caprifoliaceae)
Source: Hortic Res. 2024 May 1;11(5):uhae077. doi: 10.1093/hr/uhae077 (PMC11109519; doi:10.1093/hr/uhae077)
Supplement: Web_Material_uhae077 [file web_material_uhae077.zip › Supplemental Data Figure S26.pdf]

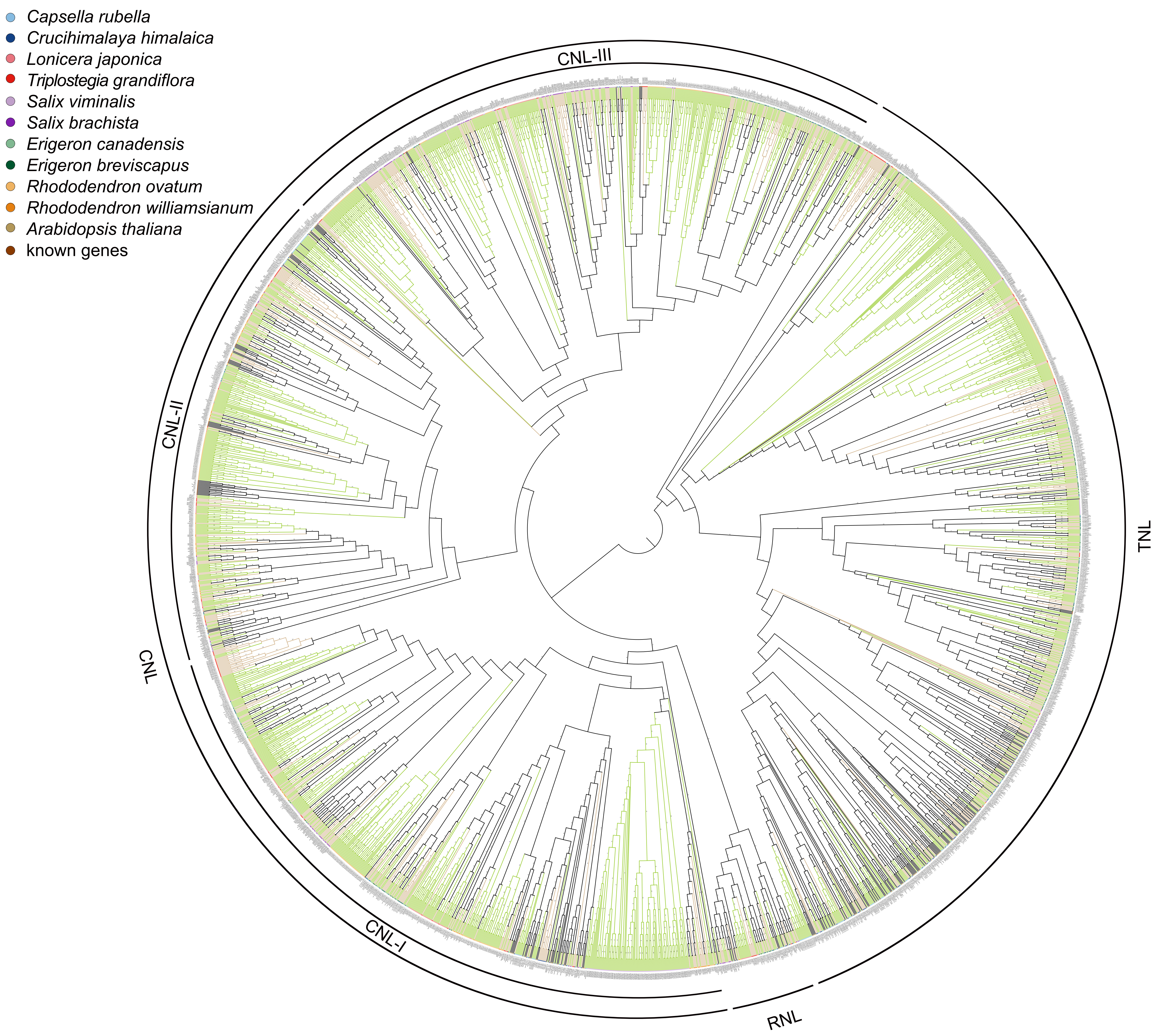

Capsella rubella
Crucihimalaya himalaica
Lonicera japonica
Triplostegia grandiflora
Salix viminalis
Salix brachista
Erigeron canadensis
Erigeron breviscapus
Rhododendron ovatum
Rhododendron williamsianum
Arabidopsis thaliana
known genes
CNL-III
CNL-II
CNL
CNL-I
RNL
TNL

Supplemental Data Figure S26. Maximum likelihood phylogenetic tree of 2,599 NBS-LRR (NLR) genes. The NLR genes of high-elevation plants (*Triplostegia grandiflora*, *Erigeron breviscapus*, *Rhododendron williamsianum*, *Crucihimalaya himalaica,* and *Salix brachista*) and their low-elevation cousins (*Lonicera japonica*, *Erigeron canadensis*, *Rhododendron ovatum*, *Capsella rubella,* and *Salix viminalis*) are represented by yellow-brown and green branches, respectively. The *Arabidopsis thaliana* NLRs and well-known functional NLRs are shown by black branches. Bootstrap support values were calculated using 1,000 replicates.
