# Supplementary material for: Coping with alpine habitats: genomic insights into the adaptation strategies of Triplostegia glandulifera (Caprifoliaceae)
Source: Hortic Res. 2024 May 1;11(5):uhae077. doi: 10.1093/hr/uhae077 (PMC11109519; doi:10.1093/hr/uhae077)
Supplement: Web_Material_uhae077 [file web_material_uhae077.zip › Supplemental Data Figure S27.pdf]

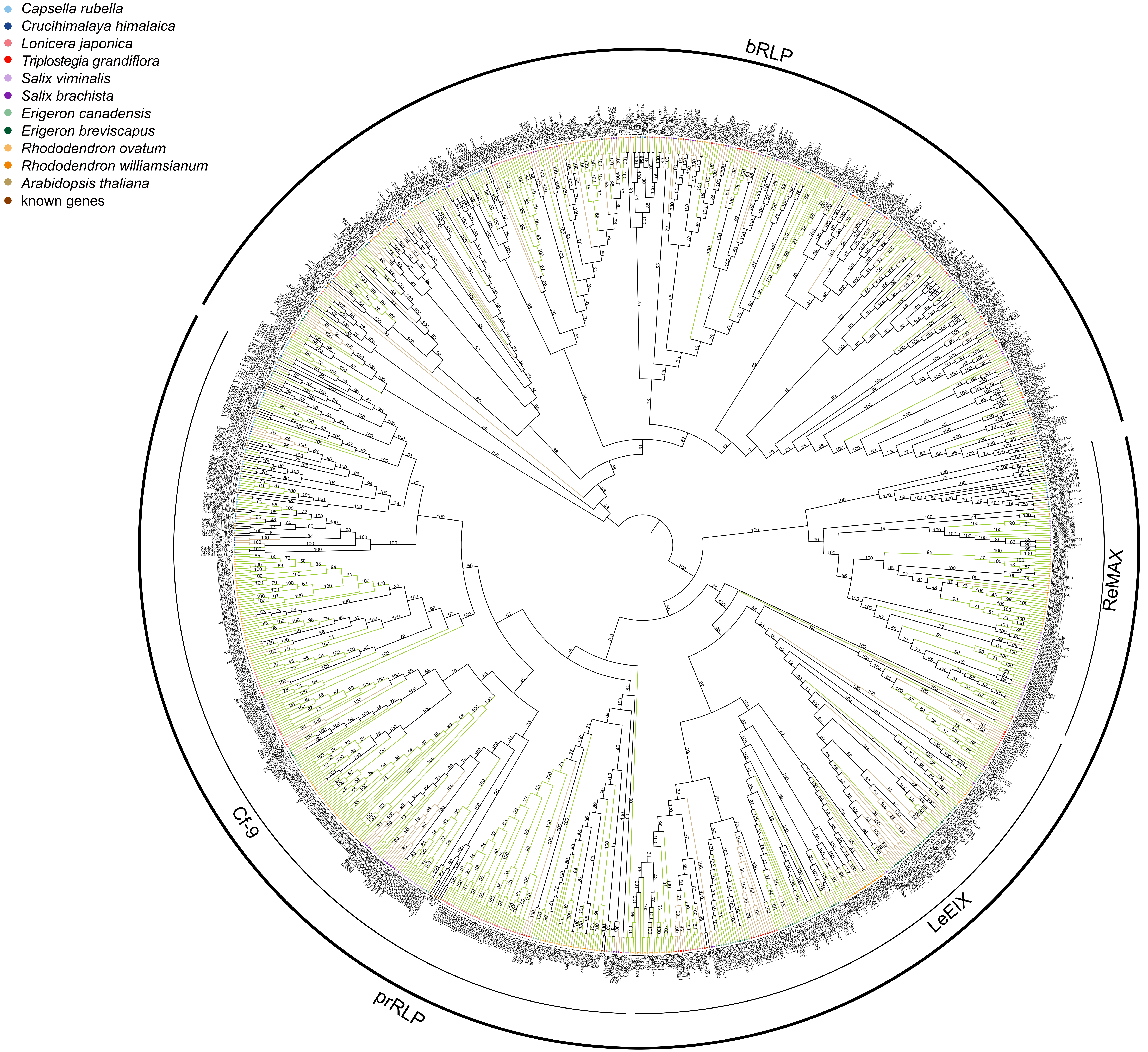

Capsella rubella
Crucihimalaya himalaica
Lonicera japonica
Triplostegia grandiflora
Salix viminalis
Salix brachista
Erigeron canadensis
Erigeron breviscapus
Rhododendron ovatum
Rhododendron williamsianum
Arabidopsis thaliana
known genes
bRLP
ReMAX
LeEIX
prRLP
Cf-9

Supplemental Data Figure S27. Maximum likelihood phylogenetic tree of 1,068 LRR-RLP (RLP) genes. The RLP genes of high-elevation plants (*Triplostegia grandiflora*, *Erigeron breviscapus*, *Rhododendron williamsianum*, *Crucihimalaya himalaica,* and *Salix brachista*) and their low-elevation cousins (*Lonicera japonica*, *Erigeron canadensis*, *Rhododendron ovatum*, *Capsella rubella,* and *Salix viminalis*) are represented by yellow-brown and green branches, respectively. The *Arabidopsis thaliana* RLPs and well-known functional RLPs are shown by black branches. Bootstrap support values were calculated using 1,000 replicates.
