# Supplementary material for: Coping with alpine habitats: genomic insights into the adaptation strategies of Triplostegia glandulifera (Caprifoliaceae)
Source: Hortic Res. 2024 May 1;11(5):uhae077. doi: 10.1093/hr/uhae077 (PMC11109519; doi:10.1093/hr/uhae077)
Supplement: Web_Material_uhae077 [file web_material_uhae077.zip › Supplemental Data Figure S28.pdf]

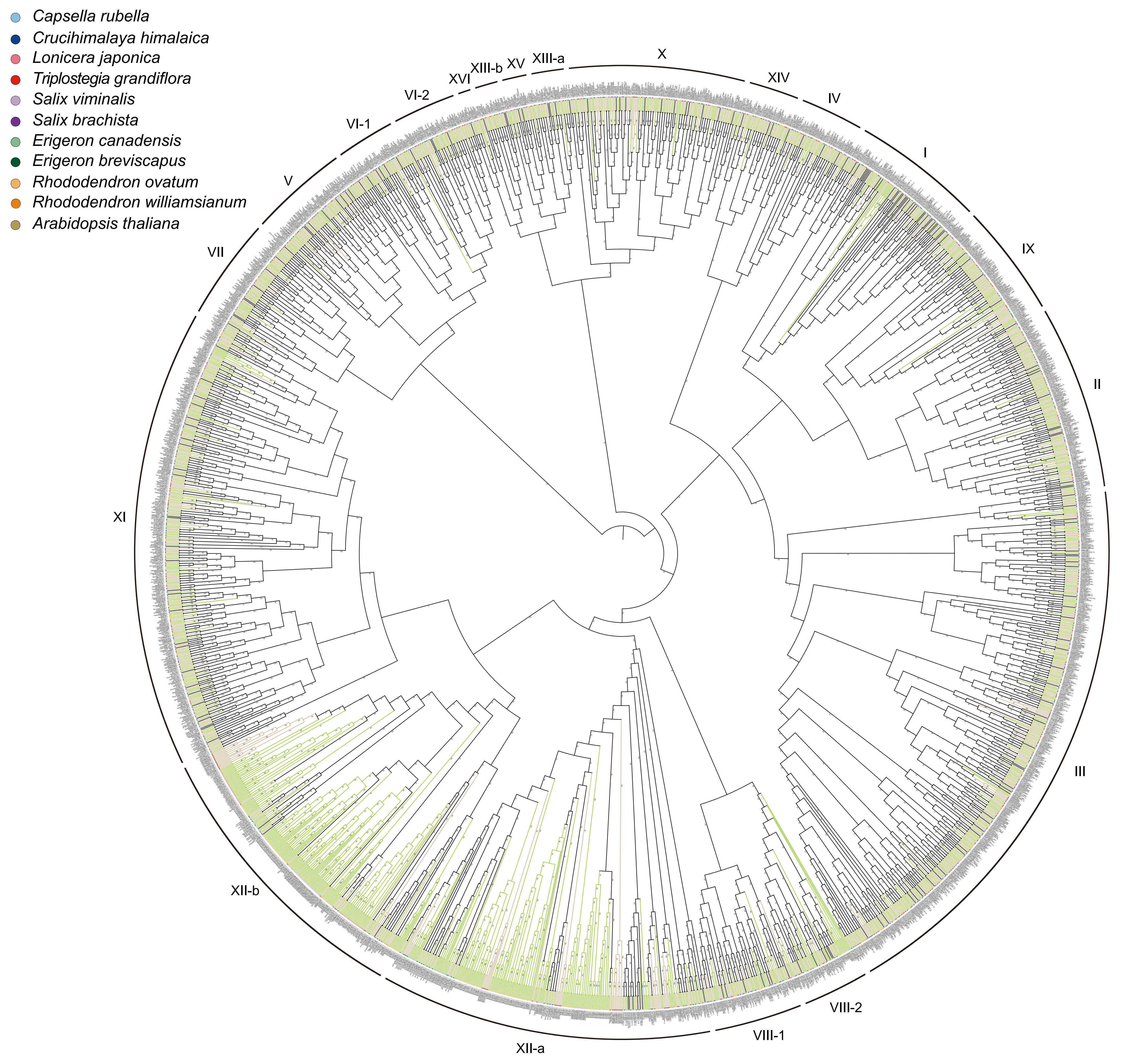
Capsella rubella
Crucihimalaya himalaica
Lonicera japonica
Triplostegia grandiflora
Salix viminalis
Salix brachista
Erigeron canadensis
Erigeron breviscapus
Rhododendron ovatum
Rhododendron williamsianum
Arabidopsis thaliana
X
XIV
IV
I
IX
II
III
VIII-2
VIII-1
XII-a
XII-b
XI
VII
V
VI-1
VI-2
XVI
XIII-b
XV
XIII-a

Supplemental Data Figure S28. Maximum likelihood phylogenetic tree of 2,376 LRR-RLK (RLK) genes. The RLK genes of high-elevation plants (*Triplostegia grandiflora*, *Erigeron breviscapus*, *Rhododendron williamsianum*, *Crucihimalaya himalaica,* and *Salix brachista*) and their low-elevation cousins (*Lonicera japonica*, *Erigeron canadensis*, *Rhododendron ovatum*, *Capsella rubella,* and *Salix viminalis*) are represented by yellow-brown and green branches, respectively. The *Arabidopsis thaliana* RLKs are shown by black branches. Bootstrap support values were calculated using 1,000 replicates.
