# Supplementary material for: Coping with alpine habitats: genomic insights into the adaptation strategies of Triplostegia glandulifera (Caprifoliaceae)
Source: Hortic Res. 2024 May 1;11(5):uhae077. doi: 10.1093/hr/uhae077 (PMC11109519; doi:10.1093/hr/uhae077)
Supplement: Web_Material_uhae077 [file web_material_uhae077.zip › Supplemental Data Figure S29.pdf]

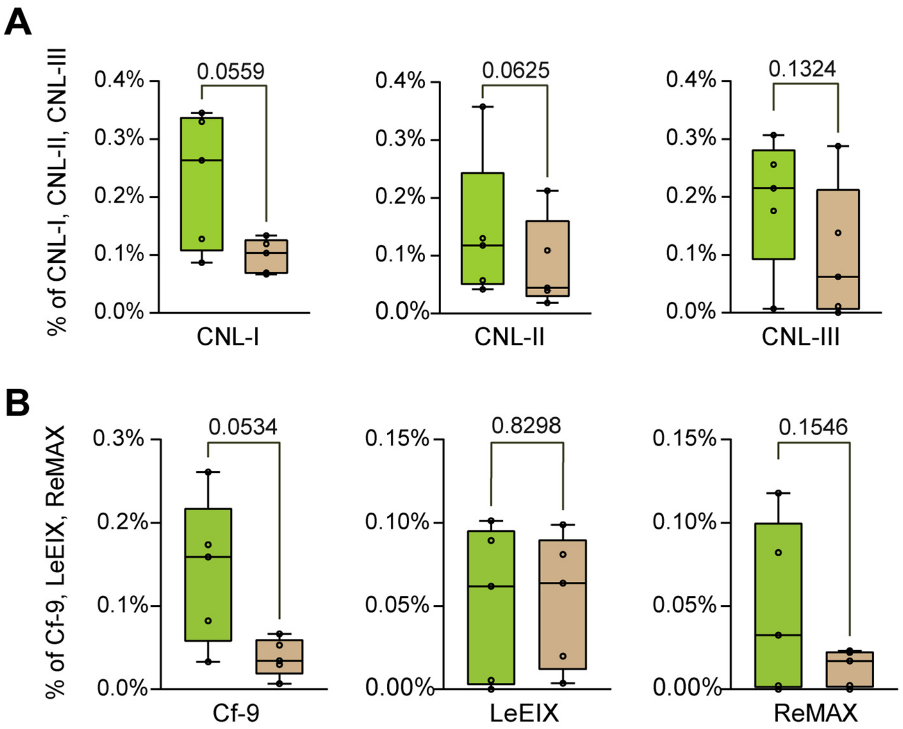

Supplemental Data Figure S29. Summary for gene percentages of subclades in NBS-LRRs (NLRs) and LRR-RLPs (RLPs). **A** Boxplots for gene percentages of CNL-I, -II, and -III subclades in NLRs in high-elevation plants (yellow-brown) compared to low-elevation plants (green). **B** Boxplots for gene percentages of Cf-9, LeEIX, and ReMAX subclades in RLPs in high-elevation plants (yellow-brown) compared to low-elevation plants (green). The percentage is the number of identified genes/number of searched genes. A paired sample t-test was used to analyze significant differences between the groups (p <0.05*).
