# Supplementary material for: Coping with alpine habitats: genomic insights into the adaptation strategies of Triplostegia glandulifera (Caprifoliaceae)
Source: Hortic Res. 2024 May 1;11(5):uhae077. doi: 10.1093/hr/uhae077 (PMC11109519; doi:10.1093/hr/uhae077)
Supplement: Web_Material_uhae077 [file web_material_uhae077.zip › Supplemental Data Figure S30.pdf]

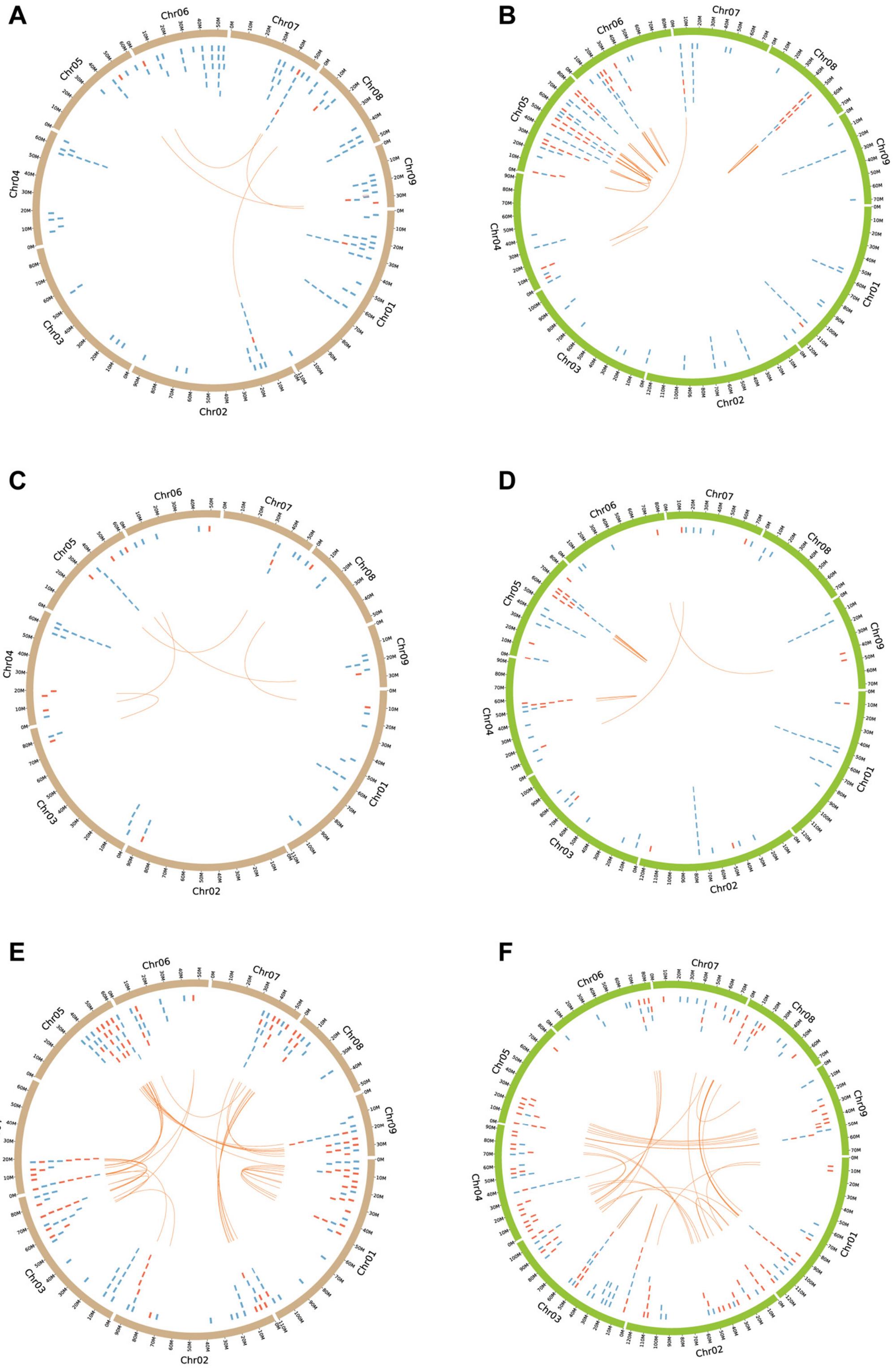

Supplemental Data Figure S30. The distributions for three types of plant immune receptors on the chromosomes of *Triplostegia glandulifera* (**A, C, E**) and *Lonicera japonica* (**B, D, F**). NLRs are shown in **A** and **B**. RLPs are shown in **C** and **D**. RLKs are shown in **E** and **F**. Lines represent syntenic gene pairs. The red vertical bars represent WGD-derived, and blue represent other duplications.
