# Supplementary material for: Coping with alpine habitats: genomic insights into the adaptation strategies of Triplostegia glandulifera (Caprifoliaceae)
Source: Hortic Res. 2024 May 1;11(5):uhae077. doi: 10.1093/hr/uhae077 (PMC11109519; doi:10.1093/hr/uhae077)
Supplement: Web_Material_uhae077 [file web_material_uhae077.zip › Supplemental Data Figure S31.pdf]

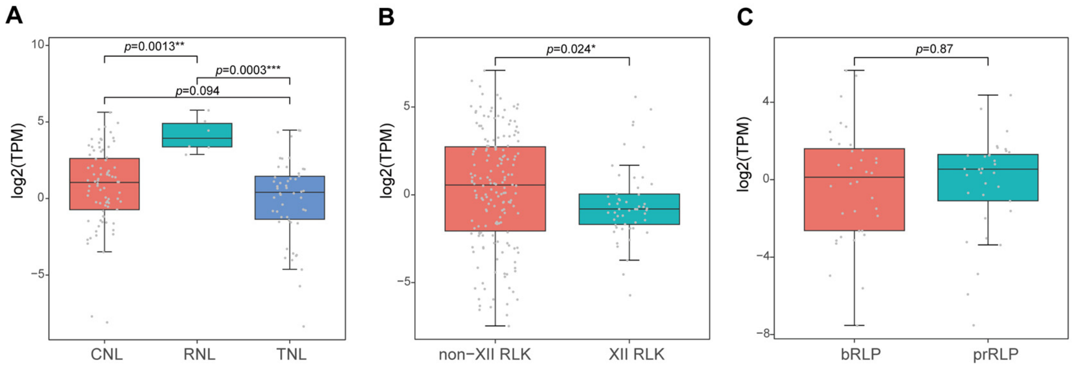

Supplemental Data Figure S31. Boxplots of gene expression levels for different clades of NLRs (**A**), RLKs (**B**), and RLPs (**C**). A wilcox test was used to analyze significant differences of expression levels between the clades ($p < 0.05$*, $p < 0.01$**, $p < 0.001$***).
