# Supplementary material for: Coping with alpine habitats: genomic insights into the adaptation strategies of Triplostegia glandulifera (Caprifoliaceae)
Source: Hortic Res. 2024 May 1;11(5):uhae077. doi: 10.1093/hr/uhae077 (PMC11109519; doi:10.1093/hr/uhae077)
Supplement: Web_Material_uhae077 [file web_material_uhae077.zip › Supplemental_Tables 1-21.docx]

**Supplemental Data Table S1.** The summary of *Triplostegia glandulifera* sequencing data.

| **Pair-end libraries** | **Insert size** | **Total data (Gb)** | **Reads length**  **(bp)** | **Sequence coverage (X)*** |
| --- | --- | --- | --- | --- |
| Illumina | 350 bp | 124.17 | 150 | 172.47 |
| PacBio | 20 kb | 98.30 | - | 136.54 |
| 10XGenomics | - | 95.89 | 150 | 133.19 |
| Hi-C | - | 149.61 | 150 | 207.81 |
| Total | - | 467.97 | - | 650.01 |

*The genome size was estimated to be 719.94 Mb by the *k*-mer analysis.

**Supplemental Data Table S2.** Statistics of different assembly versions for *Triplostegia glandulifera*.

| **Assembly versions** | **Strategy** | **Assemblied genome size (Mb)** | **Sequence number** | **N50 (Mb)** | **Max. length (Mb)** |
| --- | --- | --- | --- | --- | --- |
| V0.1 | PacBio + Illumina | 698.97 | 2,247 | 1.87 | 15.42 |
| V0.2 | V0.1+10XGenomics | 680.31 | 1,735 | 1.93/3.78* | 40.34 |
| V0.3 | V0.2+Hi-C | 680.38 | 1,233 | 1.81/66.68* | 110.59 |

N50: shortest sequence length at 50% of the genome. *statistics for contigs/scaffolds.

**Supplemental Data Table S3.** Summary of the final genome assembly for *Triplostegia glandulifera*.

|  | **Contig** | |  | **Scaffold** | |
| --- | --- | --- | --- | --- | --- |
|  | **Size (bp)** | **Number** |  | **Size (bp)** | **Number** |
| N50 | 1,814,558 | 95 |  | 66,682,622 | 4 |
| N60 | 1,361,271 | 138 |  | 61,371,989 | 5 |
| N70 | 992,416 | 196 |  | 55,562,297 | 6 |
| N80 | 547,312 | 289 |  | 53,117,383 | 8 |
| N90 | 100,735 | 561 |  | 36,352,223 | 9 |
| Longest (bp) | 15,424,425 |  |  | 110,584,275 |  |
| Total size (bp) | 678,671,803 | 2,138 |  | 680,376,494 | 1,233 |
| Total number (>=2 kb) |  | 2,132 |  |  | 1,233 |

**Supplemental Data Table S4.** The pseudomolecules length distribution for *Triplostegia glandulifera*.

| **Group** | **Sequence Number** | **Sequence Length (bp)** |
| --- | --- | --- |
| Group 1 | 71 | 110,584,275 |
| Group 2 | 114 | 94,791,608 |
| Group 3 | 57 | 88,032,427 |
| Group 4 | 77 | 66,682,622 |
| Group 5 | 73 | 61,371,989 |
| Group 6 | 36 | 55,562,297 |
| Group 7 | 73 | 54,318,866 |
| Group 8 | 103 | 53,117,383 |
| Group 9 | 82 | 36,352,223 |
| Total | 686 | 620,813,690 (91.25%) |

**Supplemental Data Table S5.** Coverage statistics of the *Triplostegia glandulifera* genome.

| **Reads** | Mapping rate (%) | 98.14 |
| --- | --- | --- |
| **Genome** | Average sequencing depth (%)* | 142.51 |
|  | Coverage (%) | 97.85 |
|  | Coverage at least 4× (%) | 96.97 |
|  | Coverage at least 10× (%) | 96.20 |
|  | Coverage at least 20× (%) | 95.29 |

*Average sequence depth indicates he average depth of each base on the genome covered by reads, and the coverage represents the proportion of genome covered by reads.

**Supplemental Data Table S6.** Summary of BUSCO assessment at the genome and protein mode for *Triplostegia glandulifera*.

|  | **BUSCO notation assessment results** |
| --- | --- |
| Genome | C:97.4% [S:92.3%, D:5.1%], F:1.2%, M:1.4%, n:1614 |
| Protein | C:95.4% [S:89.9%, D:5.5%], F:3.0%, M:1.6%, n: 1614 |

**Supplemental Data Table S7.** Transcriptome sequencing data and genome mapping for *Triplostegia glandulifera*.

| **Sample name** | **Total reads** | **Total mapped** | **Multiple mapped** | **Uniquely mapped** |
| --- | --- | --- | --- | --- |
| flower | 42,937,046 | 41,252,030 (96.08%) | 1,854,951 (4.32%) | 39,397,079 (91.76%) |
| leaf | 39,022,792 | 36,791,699 (94.28%) | 2,012,003 (5.16%) | 34,779,696 (89.13%) |
| root | 45,251,574 | 42,505,847 (93.93%) | 2,044,992 (4.52%) | 40,460,855 (89.41%) |
| stem | 36,096,882 | 33,777,647 (93.57%) | 1,438,493 (3.99%) | 32,339,154 (89.59%) |

**Supplemental Data Table S8.** Prediction of the repeat sequences for *Triplostegia glandulifera*.

| **Type** | **Repeat size (bp)** | **% of genome** |
| --- | --- | --- |
| TRF | 59,801,355 | 8.79 |
| RepeatMasker | 379,500,660 | 55.78 |
| RepeatProteinMask | 146,458,985 | 21.53 |
| Total* | 427,798,618 | 62.88 |

*Total repeat regions were identified combining all the repeat identified. As there are some overlaps between different methods, the total region is shorter than the sum of repeats identified by all the methods.

**Supplemental Data Table S9.** Categories of transposable elements (TEs) for *Triplostegia glandulifera*.

| **Type** | ***Denovo*+Repbase TEs** | | **TE proteins** | | **Combined TEs** | |
| --- | --- | --- | --- | --- | --- | --- |
|  | **Length**  **(bp)** | **% in genome** | **Length (bp)** | **% in genome** | **Length**  **(bp)** | **% in genome** |
| DNA | 23,422,454 | 3.44 | 16,029,328 | 2.36 | 34,341,906 | 5.05 |
| LINE | 5,747,472 | 0.84 | 5,147,172 | 0.76 | 8,935,909 | 1.31 |
| SINE | 60,306 | 0.01 | 0 | 0 | 60,306 | 0.01 |
| LTR | 331,440,141 | 48.71 | 125,826,402 | 18.49 | 354,414,031 | 52.09 |
| Unknown | 13,075,553 | 1.92 | 0 | 0 | 13,075,553 | 1.92 |
| Total | 379,500,660 | 55.78 | 146,458,985 | 21.53 | 412,411,535 | 60.62 |

Note: *De novo* +Repbase TEs: the result of RepeatMasker program using the repeat library predicted by *de novo* methods against the Repbase database; TE proteins: the result of RepeatProteinMask program based on known TE-related protein of Repbase; Combined TEs involve a combination of the above two methods.

**Supplemental Data Table S10.** Gene annotation summary for *Triplostegia glandulifera*.

| **Gene set** | | **Number** | **Average transcript length (bp)** | **Average CDS length (bp)** | **Average exon per gene** | **Average exon length (bp)** | **Average intron per gene** | **Average intron length (bp)** |  |
| --- | --- | --- | --- | --- | --- | --- | --- | --- | --- |
| *De novo* | AUGUSTUS | 49,199 | 2,443.66 | 1,044.39 | 3.69 | 282.69 | 2.69 | 519.32 |  |
|  | GlimmerHMM | 82,778 | 6,431.24 | 675.25 | 2.68 | 252.42 | 1.68 | 3,436.24 |  |
|  | SNAP | 84,317 | 4,018.24 | 693.69 | 3.79 | 183.24 | 2.79 | 1,193.45 |  |
|  | GeneID | 59,106 | 4,006.43 | 884.21 | 4.00 | 221.07 | 3 | 1,040.85 |  |
|  | Genscan | 42,668 | 9,019.57 | 1,279.31 | 5.55 | 230.71 | 4.55 | 1,702.96 |  |
| Homolog | *Arabidopsis thaliana* | 49,416 | 1,711.82 | 843.31 | 2.72 | 310.32 | 1.72 | 505.65 |  |
|  | *Daucus carota* | 22,210 | 4,538.18 | 1,617.42 | 4.14 | 390.86 | 3.14 | 930.74 |  |
|  | *Helianthus annuus* | 22,806 | 3,298.61 | 1,563.38 | 4.07 | 383.98 | 3.07 | 564.93 |  |
|  | *Ipomoea nil* | 19,579 | 3,811.06 | 1,532.75 | 4.15 | 369.05 | 3.15 | 722.53 |  |
|  | *Olea europaea* | 40,980 | 2,134.52 | 1,067.87 | 2.88 | 370.73 | 1.88 | 567.24 |  |
|  | *Solanum lycopersicum* | 44,583 | 2,126.25 | 1,067.07 | 2.94 | 362.36 | 1.94 | 544.62 |  |
| RNA-Seq | Cufflinks | 47,662 | 5,453.15 | 2,035.79 | 6.32 | 322.30 | 5.32 | 642.79 |  |
|  | PASA | 70,745 | 3,030.02 | 1,011.13 | 5.04 | 200.48 | 4.04 | 499.30 |  |
| EVM | | 54,450 | 2,578.08 | 986.19 | 3.69 | 267.46 | 2.69 | 592.38 |  |
| Pasa-update | | 54,193 | 2,568.68 | 991.67 | 3.68 | 269.15 | 2.68 | 587.45 |  |
| Final set | | 32,123 | 3,351.01 | 1,170.43 | 4.74 | 247.18 | 3.74 | 583.81 |  |

**Supplemental Data Table S11.** Number of predicted protein-coding genes with homology or functional classification by different methods for *Triplostegia glandulifera*.

|  | **Database** | **Number** | **Percent (%)** |
| --- | --- | --- | --- |
| Annotated | Swissprot | 21,082 | 65.63 |
|  | TrEMBL | 30,600 | 95.26 |
|  | Pfam | 24,231 | 75.43 |
|  | InterPro | 24,540 | 76.39 |
|  | GO | 14,554 | 45.31 |
|  | KO | 13,244 | 41.23 |
|  | Total | 30,688 | 95.53 |
| Unannotated |  | 1,435 | 4.47 |
| Total |  | 32,123 | 100.00 |

Gene Ontology (GO) terms and KEGG Orthology (KO) terms were provided by the blast result from EggNOG.

**Supplemental Data Table S12.** Identification of non-coding RNA genes for *Triplostegia glandulifera*.

| **Type** |  | **Copy** | **Average length (bp)** | **Total length (bp)** | **% of genome** |
| --- | --- | --- | --- | --- | --- |
| miRNA | | 1,427 | 106.10 | 151,400 | 0.022252 |
| tRNA | | 755 | 74.83 | 56,498 | 0.008304 |
| rRNA | 18S | 331 | 1,226.88 | 406,098 | 0.059687 |
|  | 28S | 865 | 138.80 | 120,064 | 0.017647 |
|  | 5.8S | 216 | 158.05 | 34,138 | 0.005018 |
|  | 5S | 5,493 | 116.63 | 640,669 | 0.094164 |
|  | Total rRNA | 6,905 | 173.93 | 1,200,969 | 0.18 |
| snRNA | CD-box | 320 | 100.63 | 32,201 | 0.004733 |
|  | HACA-box | 54 | 129.59 | 6,998 | 0.001029 |
|  | splicing | 178 | 146.95 | 26,157 | 0.003844 |
|  | Total snRNA | 552 | 118.40 | 65,356 | 0.009606 |

**Supplemental Data Table S13.** Transcriptome data of 15 species from Dipsacales.

| Family | Genus | Species | Accession number | References |
| --- | --- | --- | --- | --- |
| Caprifoliaceae | *Dipsacus* | *Dipsacus fullonum* | CRX781458 | This study |
|  | *Scabiosa* | *Scabiosa tschiliensis* | CRX781463 | This study |
|  | *Valeriana* | *Valeriana officinalis* | CRX781453 | This study |
|  | *Nardostachys* | *Nardostachys jatamansi* | SRX7804715 | Dhiman *et al*., 2020 |
|  | *Patrinia* | *Patrinia scabiosifolia* | CRX781462 | This study |
|  | *Dipelta* | *Dipelta floribunda* | CRX781456 | This study |
|  | *Kolkwitzia* | *Kolkwitzia amabilis* | CRX781454 | This study |
|  | *Abelia* | *Abelia chinensis* | CRX781452 | This study |
|  | *Diabelia* | *Diabelia spathulata* | DRX054302 | Zhao *et al*., 2017 |
|  | *Zabelia* | *Zabelia biflora* | CRX781455 | This study |
|  | *Morina* | *Morina kokonorica* | CRX781464 | This study |
|  | *Symphoricarpos* | *Symphoricarpos orbiculatus* | CRX781459 | This study |
|  | *Weigela* | *Weigela coraeensis* | CRX781457 | This study |
| Adoxaceae | *Sambucus* | *Sambucus williamsii* | CRX781461 | This study |
|  | *Viburnum* | *Viburnum opulus* | CRX781460 | This study |

**Supplemental Data Table S14.** Orthogroup clustering for *Triplostegia glandulifera* and 17 other species.

| **Species** | **Genes** | **Unclustered**  **genes** | **Genes**  **in families** | **Gene families** | **Genes**  **in unique families** | **Unique families** |
| --- | --- | --- | --- | --- | --- | --- |
| *D. fullonum* | 22,295 | 622 | 21,673 | 11,979 | 66 | 27 |
| *S. tschiliensis* | 31,230 | 1,488 | 29,742 | 15,135 | 304 | 131 |
| *T. glandulifera* | 32,123 | 1,493 | 30,630 | 14,049 | 929 | 236 |
| *V. officinalis* | 19,723 | 779 | 18,944 | 11,591 | 102 | 39 |
| *N. jatamansi* | 19,815 | 816 | 18,999 | 11,521 | 65 | 30 |
| *P. scabiosifolia* | 31,106 | 2,190 | 28,916 | 15,895 | 792 | 325 |
| *D. floribunda* | 23,817 | 1,441 | 22,376 | 13,320 | 353 | 134 |
| *K. amabilis* | 27,537 | 1,170 | 26,367 | 15,520 | 360 | 154 |
| *A. chinensis* | 18,548 | 553 | 17,995 | 11,287 | 33 | 16 |
| *D. spathulata* | 29,860 | 2,229 | 27,631 | 14,273 | 214 | 92 |
| *Z. biflora* | 22,342 | 631 | 21,711 | 12,800 | 36 | 16 |
| *M. kokonorica* | 18,130 | 595 | 17,535 | 11,002 | 57 | 26 |
| *L. japonica* | 33,939 | 1,592 | 32,347 | 13,035 | 1676 | 414 |
| *S. orbiculatus* | 21,232 | 528 | 20,704 | 12,836 | 36 | 14 |
| *W. coraeensis* | 21,665 | 597 | 21,068 | 12,976 | 43 | 18 |
| *S. williamsii* | 19,162 | 1,035 | 18,127 | 11,501 | 156 | 62 |
| *V. opulus* | 21,953 | 803 | 21,150 | 12,988 | 96 | 42 |
| *D. carota* | 31,707 | 2,388 | 29,319 | 12,981 | 4,427 | 857 |

**Supplemental Data Table S15.** MAPS result for placements of WGDs.

| MRCA | Non-duplication  Percenge | Duplication  Percentage | Non-duplication | Duplication | Total |
| --- | --- | --- | --- | --- | --- |
| N1 | 70.84% | 29.16% | 3,559 | 1,465 | 5,024 |
| N2 | 93.90% | 6.10% | 3,836 | 249 | 4,085 |
| N3 | 95.14% | 4.86% | 2,308 | 118 | 2,426 |
| N4 | 95.61% | 4.39% | 1,917 | 88 | 2,005 |
| N5 | 95.85% | 4.15% | 763 | 33 | 796 |
| N6 | 84.16% | 15.84% | 2,179 | 410 | 2,589 |

The 6,384 filtered gene families that contained at least one gene copy from each species were retained for phylogenetic tree construction. The gene trees were mapped to the species tree by the MAPS tool to calculate the percentage of subtrees with gene duplications shared among a set of species.

**Supplemental Data Table S16.** The estimated times of WGD events in Dipsacales.

|  | WGD time | up_time | down_time |
| --- | --- | --- | --- |
| Dipsacales-specific WGD | 91.97 | 87.95 | 94.71 |
| *V. officinalis*-specific WGD | 15.69 | 8.99 | 21.26 |
| *D. fullonum* and *S. tschiliensis* WGD | 28.75 | 17.04 | 37.30 |
| *Z. biflora*-specific WGD | 53.55 | 38.51 | 69.73 |
| *M. kokonorica*-specific WGD | 28.31 | 20.36 | 36.86 |

**Supplemental Data Table S17.** The sample information for RNA-seq differential expression analysis.

| Species | Number of reads | Number of mapped reads | Mapping rate | Accession number | References |
| --- | --- | --- | --- | --- | --- |
| *Triplostegia glandulifera* | 19,355,382 | 17,530,169 | 90.57% | CRX915596 | This study |
|  | 19,124,684 | 17,655,908 | 92.32% | CRX915597 |  |
|  | 23,878,969 | 21,648,673 | 90.66% | CRX915598 |  |
| *Lonicera japonica* | 29,190,651 | 27,547,217 | 94.37% | SRR22373208 | Zhang *et al*., 2022 |
|  | 22,033,699 | 20,903,370 | 94.87% | SRR22373209 |  |
|  | 24,285,050 | 23,012,513 | 94.76% | SRR22373210 |  |

**Supplemental Data Table S18.** The up-expressed genes in *Triplostegia glandulifera* compared to *Lonicera japonica* with potential functions in alpine adaptation.

|  |  |  |  |  |  |  |
| --- | --- | --- | --- | --- | --- | --- |
|  | Genes | *Triplostegia glandulifera* | *Lonicera japonica* | Log2(Fold Change) | -Log10(FDR) | *p*-value |
| Response to cold | *CBF* | TgChr04G13930.1 | Lj1C991T9.1 | 12.29 | 38.61 | 4.08E-41 |
|  | *CBF* | TgChr05G19661.1 | Lj8C30T13.1 | 7.64 | 31.10 | 2.21E-33 |
|  | *CZF1* | TgChr05G19952.1 | Lj8C66T12.1 | 4.23 | 21.07 | 4.84E-23 |
|  | *CBL1* | TgChr03G10520.1 | Lj8A278T24.1 | 3.88 | 9.99 | 1.65E-11 |
|  | *CIPK7* | TgChr08G27341.1 | Lj6A653T75.1 | 4.85 | 33.57 | 6.33E-36 |
|  | *BBX7/8* | TgChr04G14961.1 | Lj1A800G35.1 | 3.93 | 8.10 | 1.61E-09 |
|  | *LEA_2* | TgChr03G13034.1 | Lj3C917T1.1 | 12.76 | 76.09 | 8.92E-80 |
|  | *LEA_2* | TgChr03G11725.1 | Lj2P930T13.1 | 9.92 | 57.30 | 3.34E-60 |
|  | *LEA_2* | TgChr08G28028.1 | Lju124C5T8.1 | 9.11 | 34.85 | 3.08E-37 |
|  | *LEA_2* | TgChr03G11313.1 | Lj2P176T20.1 | 8.52 | 63.24 | 2.70E-66 |
|  | *LEA_2* | TgChr03G13028.1 | Lj3C916T7.1 | 7.22 | 3.04 | 4.13E-04 |
|  | *LEA_2* | TgChr07G24929.1 | Lj4A770T65.1 | 6.05 | 12.79 | 1.91E-14 |
|  | *LEA_2* | TgChr09G29292.1 | Lj4C249T6.1 | 5.76 | 15.12 | 7.00E-17 |
|  | *LEA_2* | TgChr06G22527.1 | Lj1P1050T22.1 | 5.59 | 16.49 | 2.66E-18 |
|  | *LEA_2* | TgChr06G22664.1 | Lj7A506T71.1 | 4.36 | 14.94 | 1.07E-16 |
|  | *LEA_2* | TgChr05G20269.1 | Lj8P114T30.1 | 2.98 | 4.86 | 4.55E-06 |
|  | *LEA_2* | TgChr06G22663.1 | Lj7P506T63.1 | 2.63 | 9.74 | 3.01E-11 |
|  | *LEA_2* | TgChr01G05814.1 | Lj5C44T23.1 | 2.55 | 4.65 | 7.62E-06 |
|  | *LEA_3* | TgChr04G15498.1 | Lj1A23T85.1 | 8.11 | 22.31 | 2.51E-24 |
|  | *SMP* | TgChr01G05268.1 | Lj9A672T96.1 | 6.13 | 3.94 | 4.34E-05 |
| Response to hypoxia | *ERF-VII* | TgChr01G05906.1 | Lj5A30T67.1 | 5.50 | 36.90 | 2.40E-39 |
|  | *ERF-VII* | TgChr02G06863.1 | Lj3A825T24.1 | 2.50 | 8.62 | 4.55E-10 |
|  | *ERF-VII* | TgChr04G15487.1 | Lj1C22T12.1 | 2.23 | 4.23 | 2.16E-05 |
|  | *ADH1* | TgChr09G28787.1 | Lj4A848T69.1 | 5.25 | 9.91 | 2.02E-11 |
|  | *ADH1* | TgChr05G18007.1 | Lj7C364G4.1 | 3.11 | 17.43 | 2.80E-19 |
| DNA repair | *UNG* | TgChr02G06656.1 | Lj9C88T6.1 | 7.04 | 21.53 | 1.58E-23 |
|  | *SRS2* | TgChr05G19834.1 | Lj8C49G2.1 | 3.00 | 5.93 | 3.27E-07 |
|  | *SMC3* | TgChr03G10267.1 | Lj2C853G1.1 | 3.92 | 20.36 | 2.67E-22 |
|  | *RAD51C* | TgChr06G22833.1 | Lj7A531T53.1 | 2.31 | 3.14 | 3.14E-04 |
|  | *PRD1* | TgChr07G24039.1 | Lj3C272T4.1 | 3.98 | 4.08 | 3.09E-05 |
|  | *POLK* | TgChr01G02072.1 | Lj5A219G36.1 | 2.81 | 10.49 | 5.02E-12 |
|  | *POL2A* | TgChr04G16308.1 | Lj2C988G5.1 | 2.60 | 11.53 | 4.01E-13 |
|  | *NUDX1* | TgChr02G06384.1 | Lj3A1022T62.1 | 4.06 | 4.60 | 8.51E-06 |
|  | *GTF2H1* | TgChr02G07298.1 | Lj3A773G17.1 | 2.72 | 13.20 | 7.17E-15 |
|  | *CHR24* | TgChr05G18495.1 | Lj2C614G7.1 | 2.64 | 10.24 | 9.02E-12 |
|  | *RAD1** | TgChr02G08093.1 | Lj8A305G45.1 | 3.20 | 20.48 | 2.02E-22 |
| Fast-evolving genes | *TDT* | TgChr06G20904.1 | Lj1A1241T64.1 | 3.05 | 3.84 | 5.51E-05 |
|  | *RTNLB8* | TgChr05G20156.1 | Lj8A99T66.1 | 2.29 | 8.17 | 1.36E-09 |
|  | *SDIR1* | TgChr05G20036.1 | Lj8A82T51.1 | 2.12 | 5.94 | 3.13E-07 |
|  | *LCAT3* | TgChr05G19711.1 | Lj8A197T40.1 | 2.20 | 8.07 | 1.74E-09 |
|  | *COP9* | TgChr01G01573.1 | Lj9A313T47.1 | 2.20 | 11.14 | 1.03E-12 |
|  | *CFL1* | TgChr08G26115.1 | Lj6C835T0.1 | 6.30 | 22.72 | 9.43E-25 |
|  | *ACR12* | TgChr03G13099.1 | Lj3C927G2.1 | 2.21 | 10.96 | 1.58E-12 |
|  | *ACO1* | TgChr07G24264.1 | Lj4A309G56.1 | 2.06 | 3.59 | 1.05E-04 |

**Supplemental Data Table S19.** Genomes of representative angiosperm species used in comparative genomics analyses.

| **ID** | **Species** | **Abbreviation** | **Reference** |
| --- | --- | --- | --- |
| Tgla | *Triplostegia glandulifera* | *T. glandulifera* | this study |
| Ljap | *Lonicera japonica* | *L. japonica* | https://bigd.big.ac.cn/search/?dbId=gwh&q=Lonicera%20japonica&page=1 |
| Dcar | *Daucus carota* | *D. carota* | https://www.ncbi.nlm.nih.gov/assembly/GCF_001625215.1/ |
| Hann | *Helianthus annuus* | *H. annuus* | https://www.ncbi.nlm.nih.gov/assembly/GCF_002127325.1/ |
| Slyc | *Solanum lycopersicum* | *S. lycopersicum* | https://phytozome.jgi.doe.gov/pz/portal.html#!info?alias=Org_Slycopersicum |
| Inil | *Ipomoea nil* | *I. nil* | https://www.ncbi.nlm.nih.gov/assembly/GCF_001879475.1/ |
| Oeur | *Olea europaea* | *O. europaea* | https://phytozome.jgi.doe.gov/pz/portal.html#!info?alias=Org_Oeuropaea_er |
| Atha | *Arabidopsis thaliana* | *A. thaliana* | https://plants.ensembl.org/Arabidopsis_thaliana/Info/Index |
| Grai | *Gossypium raimondii* | *G. raimondii* | https://www.ncbi.nlm.nih.gov/assembly/GCF_000327365.1 |
| Osat | *Oryza sativa* | *O. sativa* | https://plants.ensembl.org/Oryza_sativa/Info/Index |
| Sbic | *Sorghum bicolor* | *S. bicolor* | https://plants.ensembl.org/Sorghum_bicolor/Info/Index |

**Supplemental Data Table S20.** Orthogroup clustering for 11 angiosperm species.

| **Species** | **Genes** | **Unclustered**  **genes** | **Genes**  **in families** | **Gene families** | **Genes**  **in unique families** | **Unique families** |
| --- | --- | --- | --- | --- | --- | --- |
| *T. glandulifera* | 32,123 | 3,280 | 28,843 | 12,735 | 173 | 17 |
| *L. japonica* | 33,939 | 5,540 | 28,399 | 11,830 | 256 | 36 |
| *D. carota* | 31,707 | 6,277 | 25,430 | 11,934 | 770 | 70 |
| *H. annuus* | 58,163 | 7,940 | 50,223 | 12,238 | 1,588 | 55 |
| *S. lycopersicum* | 32,837 | 6,708 | 26,129 | 12,546 | 393 | 63 |
| *I. nil* | 35,123 | 2,216 | 32,907 | 12,061 | 455 | 26 |
| *O. europaea* | 50,373 | 12,701 | 37,672 | 12,376 | 698 | 47 |
| *A. thaliana* | 26,869 | 4,207 | 22,662 | 11,675 | 528 | 59 |
| *G. raimondii* | 35,226 | 2,985 | 32,241 | 12,298 | 187 | 24 |
| *O. sativa* | 34,227 | 11,580 | 22,647 | 12,395 | 84 | 34 |
| *S. bicolor* | 20,451 | 3,826 | 16,625 | 7,723 | 178 | 28 |

**Supplemental Data Table S21.** Genomes of pairs of high- vs low-elevation plants from five families.

| Scientific name | Genes | Family | Data sources | Elevation (m) |
| --- | --- | --- | --- | --- |
| *Lonicera japonica* | 33,939 | Caprifoliaceae | https://bigd.big.ac.cn/gwh/Assembly/660/show | 400–1,600 |
| *Triplostegia grandiflora* | 32,123 | Caprifoliaceae | this study | 1,500–4,000 |
| *Erigeron canadensis* | 45,426 | Asteraceae | https://bioinformatics.psb.ugent.be/plaza/versions/plaza_v5_dicots/download/download | 600–900 |
| *Erigeron breviscapus* | 43,514 | Asteraceae | http://medicinalplants.ynau.edu.cn/genome/detail/0?species=Erigeron%20breviscapus | 1,200–3,500 |
| *Rhododendron ovatum* | 41,392 | Ericaceae | http://bioinfor.kib.ac.cn/RPGD/download_genome.html | below 1,000 |
| *Rhododendron williamsianum* | 23,548 | Ericaceae | http://bioinfor.kib.ac.cn/RPGD/download_genome.html | 1,800–2,800 |
| *Capsella rubella* | 27,682 | Brassicaceae | https://phytozome-next.jgi.doe.gov/info/Crubella_v1_1 | 800–1,800 |
| *Crucihimalaya himalaica* | 27,019 | Brassicaceae | https://bioinformatics.psb.ugent.be/gdb/cruhi/ | 2,600–4,400 |
| *Salix viminalis* | 36,490 | Salicaceae | https://www.ebi.ac.uk/ena/browser/text-search?query=Salix%20viminalis | 300–1,250 |
| *Salix brachista* | 30,209 | Salicaceae | https://bigd.big.ac.cn/gwh/Assembly/663/ | 2,600–3,900 |
