# Supplementary figures and images for: Coping with alpine habitats: genomic insights into the adaptation strategies of Triplostegia glandulifera (Caprifoliaceae)
Source: Hortic Res. 2024 May 1;11(5):uhae077. doi: 10.1093/hr/uhae077 (PMC11109519; doi:10.1093/hr/uhae077)

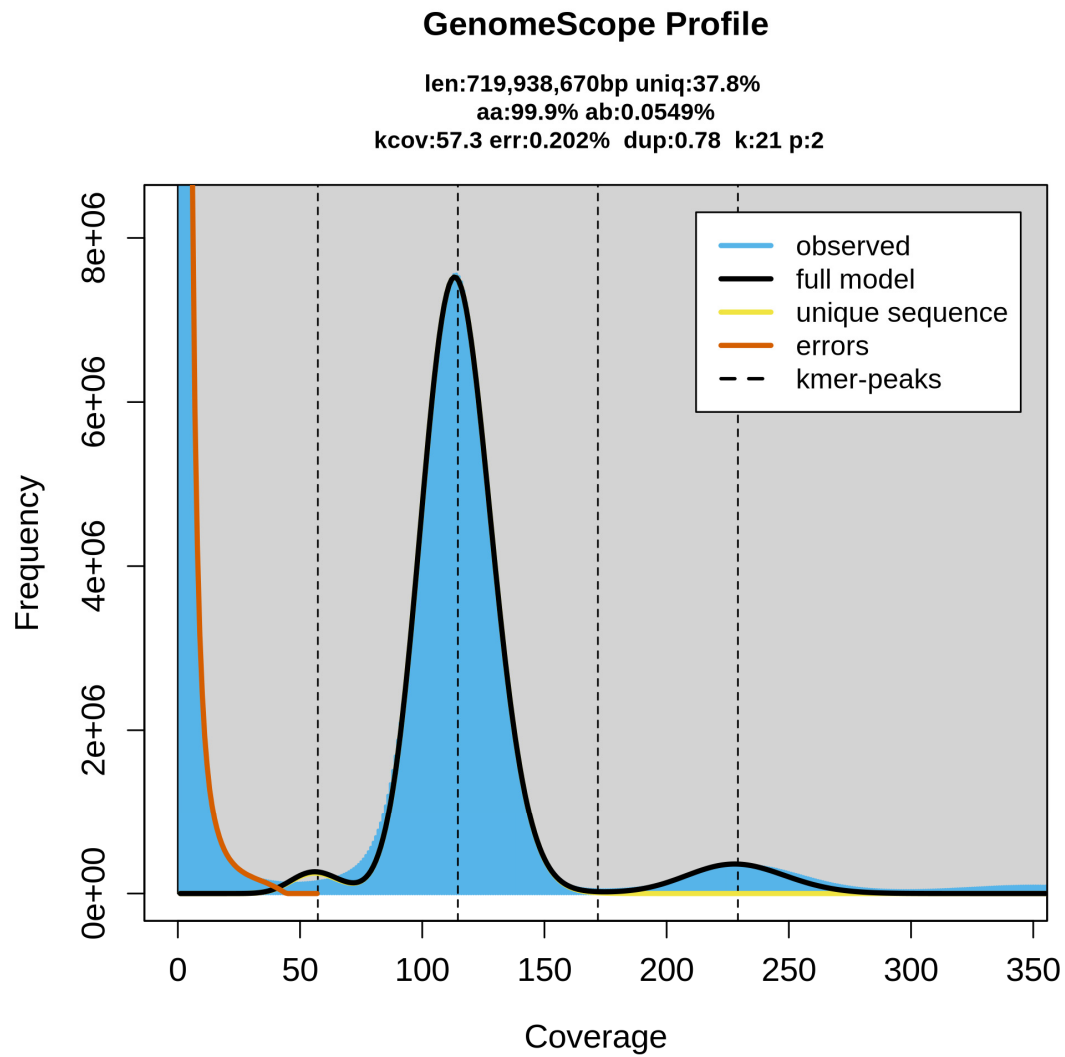

**Supplemental Data Figure S1.** Genome size estimation by GenomeScope. *K*-mer size was set as 21.

Supplement: Web_Material_uhae077 [file web_material_uhae077.zip › Supplemental Data Figure S1.pdf]

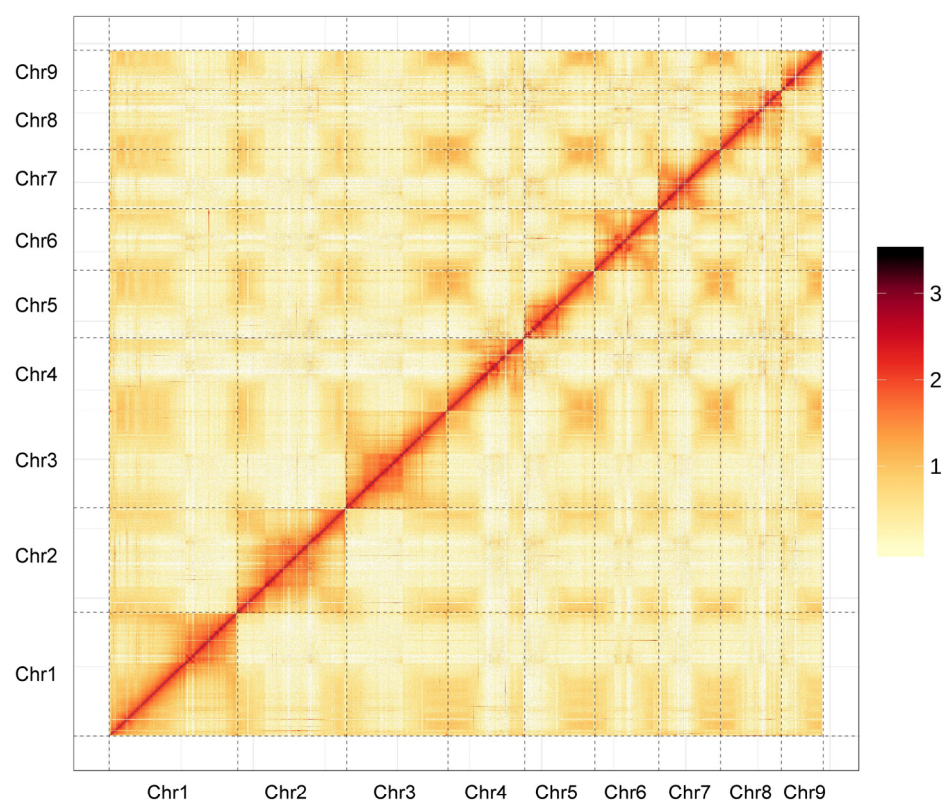

**Supplemental Data Figure S3.** Hi-C intra-chromosomal contact map (2n=18) of *Triplostegia glandulifera*.

Supplement: Web_Material_uhae077 [file web_material_uhae077.zip › Supplemental Data Figure S3.pdf]
