# Supplementary material for: Coping with alpine habitats: genomic insights into the adaptation strategies of Triplostegia glandulifera (Caprifoliaceae)
Source: Hortic Res. 2024 May 1;11(5):uhae077. doi: 10.1093/hr/uhae077 (PMC11109519; doi:10.1093/hr/uhae077)
Supplement: Web_Material_uhae077 [file Supplemental Data Figure S12.pdf › Supplemental Data Figure S12.pdf]

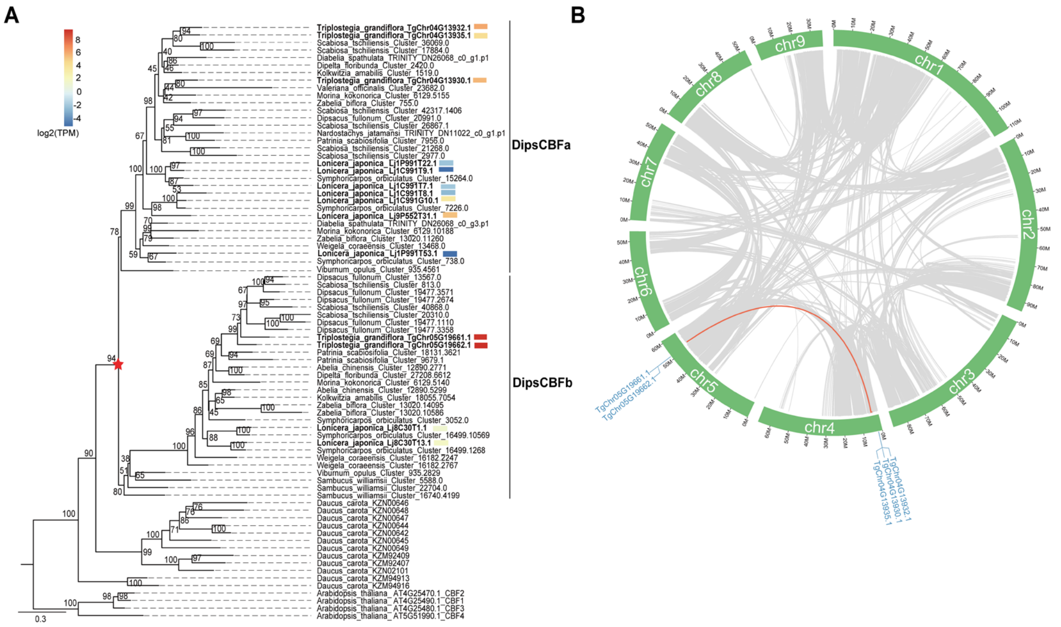

Supplemental Data Figure S12. The evolution of *CBF* genes in Dipsacales. **A** Phylogenetic tree of *CBF* genes in Dipsacales. The expression level of each *CBF* gene in *Triplostegia glandulifera* and *Lonicera japonica* was shown next to the gene ID. The *CBF* genes from *Daucus carota* are used as reference, and *CBF* genes from *Arabidopsis thaliana* as outgroup. Bootstrap support values were calculated using 1,000 replicates. The red asterisk indicates the duplication event. **B** The circos map of *CBF* genes in the *T. glandulifera* genome. The red line links the syntenic gene and the blue color labels the tandem duplicate copies.
